# P21-activated kinase 1 (PAK1)-mediated cytoskeleton rearrangement promotes SARS-CoV-2 entry and ACE2 autophagic degradation

**DOI:** 10.1038/s41392-023-01631-0

**Published:** 2023-10-09

**Authors:** Ming Liu, Bingtai Lu, Yue Li, Shuofeng Yuan, Zhen Zhuang, Guangyu Li, Dong Wang, Liuheyi Ma, Jianheng Zhu, Jinglu Zhao, Chris Chung-Sing Chan, Vincent Kwok-Man Poon, Kenn Ka-Heng Chik, Zhiyao Zhao, Huifang Xian, Jingxian Zhao, Jincun Zhao, Jasper Fuk-Woo Chan, Yuxia Zhang

**Affiliations:** 1 Guangzhou Institute of Pediatrics, Guangzhou Women and Children’s Medical Centre, State Key Laboratory of Respiratory Diseases, Guangzhou Medical University, 510623 Guangzhou, Guangdong China; 2 Medical Research Institute, Guangdong Provincial People’s Hospital (Guangdong Academy of Medical Sciences), Southern Medical University, Guangdong, China; 3 State Key Laboratory of Emerging Infectious Diseases, Carol Yu Centre for Infection, Department of Microbiology, School of Clinical Medicine, Li Ka Shing Faculty of Medicine, The University of Hong Kong, Pokfulam, Hong Kong China; 4 Department of Clinical Microbiology and Infection Control, The University of Hong Kong-Shenzhen Hospital, Shenzhen, Guangdong China; 5 Centre for Virology, Vaccinology and Therapeutics, Hong Kong Science and Technology Park, Hong Kong, China; 6 State Key Laboratory of Respiratory Disease, National Clinical Research Center for Respiratory Disease, Guangzhou Institute of Respiratory Health, the First Affiliated Hospital of Guangzhou Medical University, Guangzhou, China; 7 The Third Affiliated Hospital of Zhengzhou University, 450052 Zhengzhou, China; 8 Guangzhou Laboratory, Guangzhou, Guangdong Province China; 9 Chongqing International Institute for Immunology, Chongqing, China

**Keywords:** Infectious diseases, Respiratory tract diseases

## Abstract

Severe acute respiratory syndrome coronavirus 2 (SARS-CoV-2), the causative agent of coronavirus disease 2019 (COVID-19), has had a significant impact on healthcare systems and economies worldwide. The continuous emergence of new viral strains presents a major challenge in the development of effective antiviral agents. Strategies that possess broad-spectrum antiviral activities are desirable to control SARS-CoV-2 infection. ACE2, an angiotensin-containing enzyme that prevents the overactivation of the renin angiotensin system, is the receptor for SARS-CoV-2. ACE2 interacts with the spike protein and facilitates viral attachment and entry into host cells. Yet, SARS-CoV-2 infection also promotes ACE2 degradation. Whether restoring ACE2 surface expression has an impact on SARS-CoV-2 infection is yet to be determined. Here, we show that the ACE2-spike complex is endocytosed and degraded via autophagy in a manner that depends on clathrin-mediated endocytosis and PAK1-mediated cytoskeleton rearrangement. In contrast, free cellular spike protein is selectively cleaved into S1 and S2 subunits in a lysosomal-dependent manner. Importantly, we show that the pan-PAK inhibitor FRAX-486 restores ACE2 surface expression and suppresses infection by different SARS-CoV-2 strains. FRAX-486-treated Syrian hamsters exhibit significantly decreased lung viral load and alleviated pulmonary inflammation compared with untreated hamsters. In summary, our findings have identified novel pathways regulating viral entry, as well as therapeutic targets and candidate compounds for controlling the emerging strains of SARS-CoV-2 infection.

## Introduction

Severe acute respiratory syndrome coronavirus 2 (SARS-CoV-2) induced coronavirus disease 2019 (COVID-19) has caused significant healthcare and economic burdens globally.^1,2^ The Omicron variant of SARS-CoV-2, characterized by striking immune evasiveness and high transmissibility, has brought new challenges to pandemic control.^3–6^ Although vaccination is highly effective in reducing the risk of severe disease and death, it is suboptimal for preventing transmission of SARS-CoV-2.^7,8^ Among patients at high risk of developing severe disease, direct virus-acting antivirals such as nirmatrelvir/ritonavir and molnupiravir are mainly effective during the first 5 days of infection before the cytokine storm-induced immunopathology is initiated.^9,10^ Moreover, these antivirals may not be tolerated in some patients due to drug interactions and side effects. Therefore, novel treatment options for COVID-19 remain a research priority.

Coronaviruses are positive single-stranded RNA viruses that belong to the family of *Coronaviridae* in the order *Nidovirales*. They infect a wide range of mammalian and avian species.^11,12^ Effective entry is a prerequisite for the virus to deliver its genomic materials into the host cells.^2^ Similar to other coronaviruses such as SARS-CoV,^13^ MERS-CoV^14^ and HCoV-HKU1,^15^ SARS-CoV-2 enters the host cell via cathepsin-mediated endosomal pathway and TMPRSS2-mediated non-endosomal pathway.^16^ For the endosomal pathway, the virus is endocytosed into the cell and fused with the endosomal and lysosomal membrane to release its genomic materials.^17^ Alternatively, the viral envelope fuses with the cell plasma membrane with the help of cleaved spike protein (S2), which makes dramatic conformational changes to tie the viral and cellular membranes together, creating a fusion pore to inject the genomic materials directly into the host cytoplasm.^18,19^ The current circulating strain Omicron relied more on the endocytic pathway than plasma membrane fusion.^20^ The spike protein is located on the viral surface and is responsible for the virus to attach to the host membrane.^21^ It is a transmembrane glycoprotein that presents as homotrimers. The spike protein can be cleaved into S1 and S2 subunits by the type II transmembrane serine protease TMPRSS2, furin or lysosomal cathepsins.^18,22^ The S1 subunit consists of a receptor binding domain (RBD) that binds to host cells, whereas the S2 subunit is a helical transmembrane region and is responsible for the fusion of the viral membrane with the host membrane.

SARS-CoV-2 attachment and entry into the host cell rely on the interactions between the spike protein and the host receptor angiotensin-converting enzyme 2 (ACE2).^23^ The main physiological role of ACE2 is to catalyze angiotensin II (AngII) into angiotensin 1-7 and prevents overactivation of the renin angiotensin system (RAS).^24^ SARS-CoV infection has been shown to decrease the protein level of ACE2,^25^ possibly by shedding of ACE2 via ADAM17-dependent cleavage.^26*–*28^ Based on the sequence and structural similarity of SARS-CoV and SARS-CoV-2,^11^ it has been suggested that SARS-CoV-2 infection would also lead to ACE2 degradation.^29*–*31^ Indeed, using a pseudotyped virus assay, it has recently been shown that SARS-CoV-2 spike protein downregulates ACE2 expression and impairs endothelial function.^32^ However, the molecular mechanism underlying ACE2 degradation and its relevance with SARS-CoV-2 infection require further exploration.

In this study, we show that SARS-CoV-2 cell entry promotes ACE2 endocytosis and degradation. The ACE2-spike complex is internalized by clathrin-mediated endocytosis and P21-activated kinase 1 (PAK1)-mediated cytoskeleton rearrangement. Subsequently, the complex is degraded by autophagy. In addition, we show that a pan-PAK inhibitor FRAX486 displays potent and broad activity in suppressing SARS-CoV-2 infection.

## Results

### SARS-CoV-2 entry into host cells promotes ACE2 degradation

**Fig. 1:**
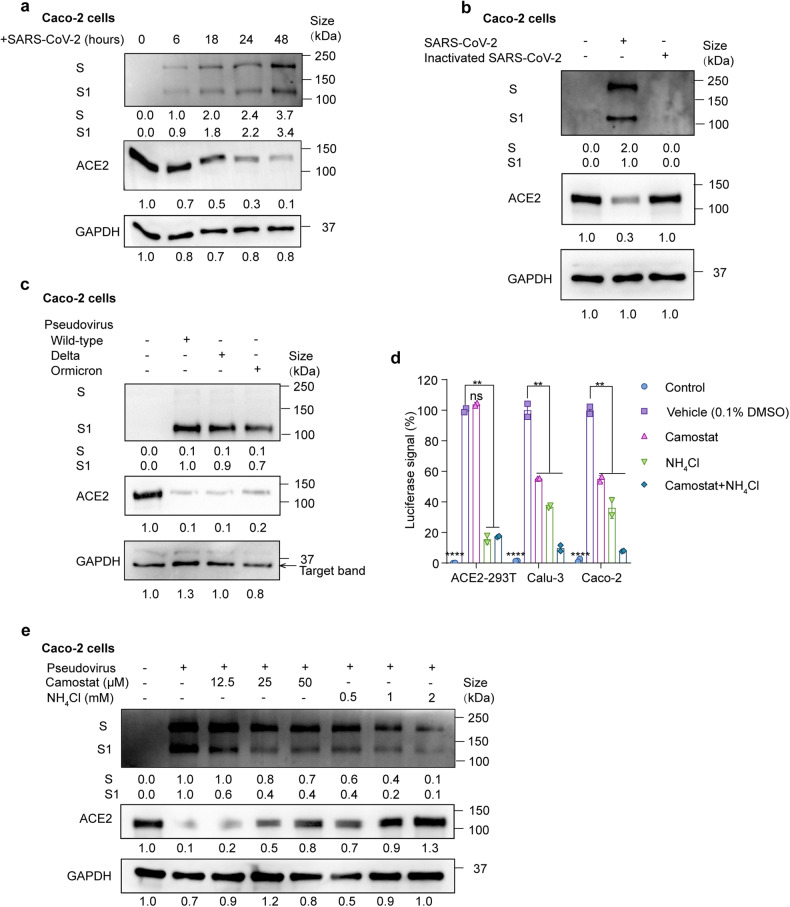
SARS-CoV-2 infection promotes ACE2 degradation. **a** Immunoblot analysis of extracts of Caco-2 cells infected by SARS-CoV-2 at 6, 18, 24 and 48 h post-infection (MOI = 0.01). S and S1 were detected using an anti-S1 antibody. **b** Immunoblot analysis of extracts of Caco-2 cells infected by SARS-CoV-2 and heat-inactivated SARS-CoV-2 at 24 h post-infection (MOI = 0.01). S and S1 were detected using an anti-S1 antibody. **c** Immunoblot analysis of extracts of Caco-2 cells infected by pseudotyped SARS-CoV-2 variants (Wild-type (WT), delta and omicron) at 24 h post-infection. S and S1 were detected using an anti-S1 antibody. **d** Luciferase signals showing entry of SARS-CoV-2 pseudovirus into three cell lines (ACE2-293T, Calu-3, Caco-2) with vehicle (H_2_O), TMPRSS2 inhibitor camostat (50 μM) or endocytosis inhibitor NH_4_Cl (2 mM) for 48 h. Uninfected cells were included as negative control. Viral entry efficiency was calculated as the percentages of luciferase signal with inhibitors/luciferase signal with vehicle (H_2_O). Error bars indicate SEM. *P*-values were calculated by Tukey’s multiple comparison test. **P < 0.01; ****P < 0.0001; ns: not significant. **e** Immunoblot analysis of extracts of Caco-2 cells infected by SARS-CoV-2 pseudovirus for 24 h with the treatment of camostat or NH_4_Cl at the indicated concentration. Unless otherwise specified, *n* = 3 biologically independent experiments were performed (**a**–**e**)

Previous studies have demonstrated that infection with SARS-CoV results in a decrease in ACE2 protein levels.^25*–*28^ Pseudotyped SARS-CoV-2 virus also downregulates ACE2 expression.^32^ Yet it remains unclear how SARS-CoV-2 induces ACE2 degradation and whether this degradation is strain-specific. We first analyzed SARS-CoV-2 (SARS-CoV-2/human/CHN/IQTC01/2020 (GenBank accession no. MT123290.1, hereafter termed wild-type or WT) infected Caco-2 cells and ACE2-293T cells (HEK-293T cells stably expressed ACE2) and found that spike protein cleavage and ACE2 degradation occurred in a time-dependent manner (Fig. 1a, Supplementary Fig. S1a,b). When incubated with the heat-inactivated SARS-CoV-2 for 24 h, ACE2 expression was not attenuated (Fig. 1b). Subsequently, in order to study SARS-CoV-2 entry in a biosafety level 2 (BSL-2) laboratory, we utilized replication-deficient lentivirus pseudotyped with the SARS-CoV-2 spike protein.^33^ SARS-CoV-2 pseudovirus infection exhibited more than a 500-fold increase in luciferase activities in ACE2-293T cells but not in 293T cells (Supplementary Fig. S1c), which is consistent with ACE2 being the host receptor for SARS-CoV-2.^18,22^ Consistent with SARS-CoV-2 infection, ACE2 degradation was also observed when infected by WT, delta and omicron pseudovirus (Fig. 1c), suggesting a common mechanism across strains in ACE2 degradation. The mRNA level of ACE2 remained unchanged when infected by pseudovirus (Supplementary Fig. S1d). This suggested that the loss of ACE2 was due to increased protein degradation. ADAM17 has been shown to shed ACE2 during SARS-CoV infection.^26*–*28^ However, in the setting of SARS-CoV-2 infection, we did not observe ACE2 in the cell culture supernatant, and ACE2 degradation was not inhibited when ADAM17 inhibitor TAPI-1 was added during pseudovirus infection (Supplementary Fig. S1e). Together, we have demonstrated that SARS-CoV-2 infection leads to ACE2 degradation at protein levels. Importantly, this degradation is dependent on viral activity and is not strain-specific.

Spike protein mediates viral entry via TMPRSS2-mediated membrane fusion and clathrin-mediated endocytosis.^18,34^ But whether viral fusion or endocytosis leads to ACE2 degradation remains unknown. Therefore, we applied camostat mesylate (TMPRSS2 inhibitor) and NH_4_Cl (inhibiting endosomal acidification and endocytosis) in non-toxic concentrations during pseudovirus infection (Supplementary Fig. S1f). Camostat mesylate reduced viral infection by about 50% in Calu-3 and Caco-2 cells but not in ACE2-293T cells, while NH_4_Cl stably inhibited viral entry in all cell lines (Fig. 1d). Since TMPRSS2 is endogenously expressed in Calu-3 and Caco-2 cells, but not in ACE2-293T cells, the TMPRSS2 expression level may explain the differences in inhibition of viral entry of camostat mesylate. Both camostat mesylate and NH_4_Cl inhibited ACE2 degradation upon pseudovirus infection in Caco-2 cells dose-dependently (Fig. 1e). These results suggest that both membrane fusion and endocytosis of SARS-CoV-2 promote ACE2 degradation.

### SARS-CoV-2 spike protein mediates ACE2 internalization and degradation

**Fig. 2:**
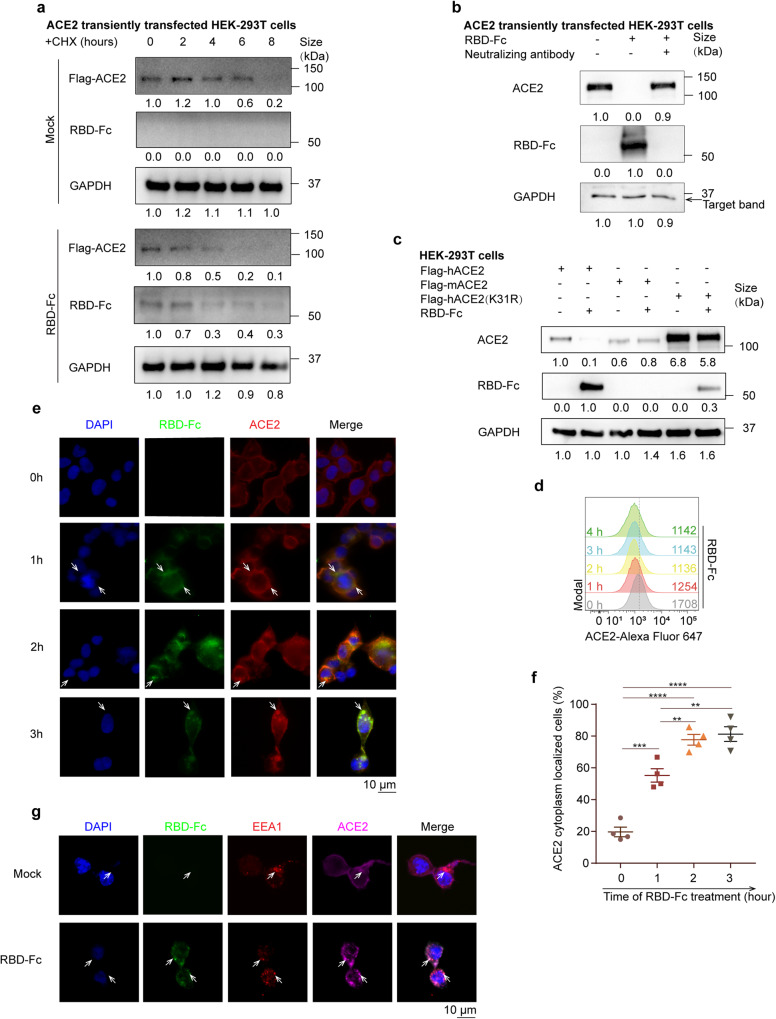
Viral spike protein mediates viral entry and ACE2 endocytosis. **a** Immunoblots of cell lysates of ACE2 transiently transfected HEK-293T cells treated with Cycloheximide (CHX) (100 μg/ml) at 2-h interval for 8 h in the presence of RBD-Fc or mock. **b** Immunoblots of extracts of ACE2 transiently transfected HEK-293T cells treated with RBD-Fc and a neutralizing antibody recognizing SARS-CoV-2 spike RBD domain as indicated. **c** Immunoblots of extracts of HEK-293T cells transfected with Flag-tagged human ACE2(Flag-hACE2), murine ACE2 (Flag-mACE2) or a K31R variant of human ACE2(Flag-hACE2(K31R)), and treated with/without RBD-Fc. **d** Flow cytometry histograms showing surface ACE2 in ACE2-HEK293T cells at indicated time points post RBD-Fc treatment. The mean fluorescent intensities are shown. **e** Immunofluorescent microscopy images showing the localization of ACE2 (red) in Flag-ACE2 transfected HEK293T cells treated with RBD-Fc (green) at indicated time points. Cell nuclei were stained with DAPI (blue). Colocalization of ACE2 and RBD-Fc were indicated by white arrows. Scale bars, 10 μm. **f** Statistic diagram of (**e**) showing percentages of ACE2 cytoplasm localized cells to total ACE2-expressing cells. Each point represents a field of view. Error bars indicate SEM (*n* = 4). *P*-values were calculated by Tukey’s multiple comparison test. **P < 0.01; ***P < 0.001. All unlabeled comparisons are not significant. **g** Immunofluorescent microscopy images showing the localization of ACE2 (magenta) and EEA1(red) in Flag-ACE2 transfected HEK293T cells treated with RBD-Fc (green) at 1 h post-treatment. Cell nuclei were stained with DAPI (Blue). Colocalization of ACE2, EEA1 and RBD-Fc are indicated by white arrows. Scale bars, 10 μm. Unless otherwise specified, *n* = 3 biologically independent experiments were performed (**a**–**g**)

SARS-CoV-2 utilizes the spike protein to bind with ACE2 for viral attachment and endocytosis. However, it is not clear whether the interaction between spike and ACE2 contributes to the degradation and internalization of ACE2. We treated ACE2 transiently transfected 293T cells with the spike protein receptor binding domain fused with a mouse Fc (RBD-Fc). Cycloheximide (CHX) was added to exclude the effect of protein synthesis. ACE2 degradation was accelerated in the presence of RBD-Fc (Fig. 2a and Supplementary Fig. S2a). Similar results were also observed using Caco-2 cells (Supplementary Fig. S2b). RBD-Fc promoted ACE2 degradation in a dose-dependent manner (Supplementary Fig. S2c). We next analyzed whether the binding of ACE2 with spike protein mediates ACE2 degradation. Disrupting ACE2-S binding by a neutralizing antibody prevents ACE2 internalization as well as ACE2 degradation (Fig. 2b). We then included ACE2 constructs that bind spike protein with different affinities. Murine ACE2 does not bind to viral spike protein.^35^ K31R is a mutant of human ACE2 that binds spike protein with lower affinity (EC50 1.01 ± 0.04 vs 298 ± 0.64 nM, WT vs K31R).^36^ RBD-Fc mediated ACE2 degradation was attenuated for mACE2 and the K31R mutant of human ACE2 (Fig. 2c). To further confirm that endocytosis promotes ACE2 degradation, we incubated ACE2-293T cells with RBD-Fc. The surface expression of ACE2 began to decrease within 1 h (Fig. 2d). Fluorescence microscopy imaging showed endocytosis of the RBD-Fc-ACE2 complex from the cell membrane to the cytoplasm (Fig. 2e, f and Supplementary Fig. S2d). ACE2 was colocalized with the early endosome marker EEA1 (Fig. 2g and Supplementary Fig. S2e), supporting that endocytosis mediates internalization of the complex. We concluded that the spike protein RBD domain alone is sufficient to induce ACE2 endocytosis and degradation, and ACE2-spike interaction is a prerequisite for ACE2 degradation.

### Autophagy-lysosome pathway mediates ACE2-spike degradation

**Fig. 3:**
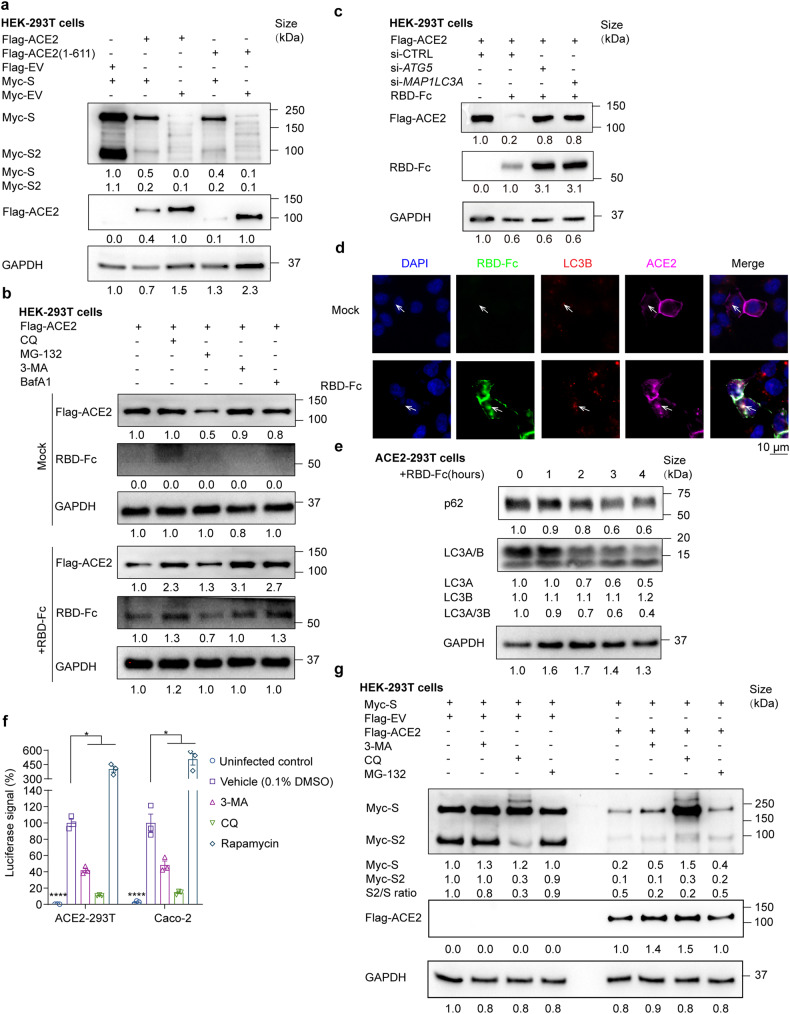
ACE2-S complex is degraded via autophagy-lysosome pathway. **a** Immunoblot analysis of extracts of HEK-293T transfected with Flag-ACE2, Flag-ACE2(1-611) or Flag-EV, in combination with Myc-S or Myc-EV. **b** Immunoblot images showing extracts of ACE2-293T cells treated with mock or RBD-Fc in the presence of autophagy inhibitors (3-MA (5 mM), CQ (25 μM) or BafA1 (0.2 μM)) or a protease inhibitor (MG132 (10 μM)). **c** Immunoblot analysis of HEK-293T extracts transfected with Flag-ACE2 and siRNA targeting *ATG5*, *MAP1LC3A* or a control siRNA(si-CTRL) for 48 h, and treated with RBD-Fc for 6 h. **d** Immunofluorescent microscopy images showing colocalization of ACE2 (magenta), LC3B (red) and RBD-Fc (green) in Flag-ACE2 transfected HEK293T cells treated with RBD-Fc at 3 h post-treatment. Cell nuclei were stained with DAPI (Blue). Colocalization of ACE2, RBD-Fc and LC3 were indicated by white arrows. Scale bars, 10 μm. **e** Immunoblot analysis of ACE2-293T cells treated with RBD-Fc at 1-h interval for 4 h. **f** Luciferase signals showing SARS-CoV-2 pseudovirus entry into ACE2-293T and Caco-2 cells with vehicle (0.1% DMSO), autophagy inhibitors (3-MA (5 mM) or CQ (25 μM)) or an autophagy promoter (rapamycin (50 μM)) at 48 h post-infection. Viral entry efficiency was calculated as the percentages of luciferase signal with inhibitors/vehicle (0.1% DMSO) treated cells. Error bars indicate SEM. *P*-values were calculated by Tukey’s multiple comparison test. *P < 0.05, ****P < 0.0001. **g** Immunoblot analysis showing extracts of HEK-293T cells transfected with Myc-S, ACE2 and/or Flag-EV with the treatment of 3-MA (10 mM), chloroquine (CQ) (50 μM) or MG132 (10 μM). Unless otherwise specified, *n* = 3 biologically independent experiments were performed (**a**–**g**)

The spike protein enhances endocytosis and degradation of ACE2, but the exact mechanism is not known. We set forth to examine whether spike protein has an impact on cytoplasmic ACE2 degradation. We co-expressed full-length ACE2 and the ectodomain of ACE2 (aa. 1-611) with spike protein. The ectodomain of ACE2 (aa. 1-611) lacked a membrane localization sequence and was exclusively localized in the cytoplasm (Supplementary Fig. S3a and S3b). Co-expression of spike protein facilitated the degradation of the full-length ACE2 and its intracellularly-expressed ectodomain (Fig. 3a). Spike protein level was also reduced when co-expressed with ACE2, implying that the two proteins were degraded in a complex (Fig. 3a). To identify how spike protein mediates ACE2 degradation, we treated ACE2 transfected 293T cells with RBD-Fc and autophagy inhibitors 3-Methyladenine (3-MA), Chloroquine (CQ) and Bafilomycin A1 (BafA1), as well as a protease inhibitor MG132. 3-MA, CQ and BafA1 restored ACE2 and RBD-Fc level but not MG132 (Fig. 3b and Supplementary Fig. S3c). Similar results were also observed using Caco-2 cells (Supplementary Fig. S3d). Knockdown of autophagy-related genes *ATG5* and *MAP1LC3A* prohibited the degradation of ACE2 and RBD-Fc, supporting that ACE2 was degraded by the autophagosome-lysosome pathway (Fig. 3c). Moreover, colocalization of ACE2, spike protein, and LC3B (a protein localized in the autophagosome membrane), was observed by immunofluorescence imaging (Fig. 3d and Supplementary Fig. S3f). The fluorescence intensity of LC3B was also enhanced when treated with RBD-Fc (Fig. 3d). To verify whether S protein entry affects autophagy flux, we incubated RBD-Fc and ACE2-293T cells and measured the autophagic marker, LC3A/B and p62. The LC3A/LC3B ratio and p62 protein decreased gradually as incubation time increased (Fig. 3e and Supplementary Fig. S3g), which suggested that S protein promoted autophagy during its entry. We then treated pseudovirus-infected cells with autophagy inhibitors (3-MA and CQ) and autophagy enhancer rapamycin to determine the role of autophagy in viral entry in non-toxic concentration determined in Supplementary Fig. S3h. Our results showed that inhibiting autophagy with 3-MA and CQ significantly decreased viral yield, while promoting autophagy with rapamycin enhanced viral entry by 5-fold, supporting that autophagy promotes SARS-CoV-2 entry (Fig. 3f). In conclusion, our study provides evidence showing that the spike protein and ACE2 undergo autophagic degradation in a complex.

It has been reported that selective cleavage of SARS-CoV-2 spike enhances viral entry.^22^ We next investigated whether ACE2 affects spike protein cleavage. Co-expression of spike with ACE2 significantly decreased the ratio of S2/S compared with co-expression with an empty vector (Fig. 3g and Supplementary Fig. S3h), demonstrating that ACE2 inhibited the selective cleavage of full-length spike protein. We reasoned that ACE2 may interact with the cleavage site between S1 and S2 and prevent its cleavage. Notably, although both CQ and 3-MA prevented autophagic degradation of full-length spike protein, only CQ inhibited spike protein cleavage (Fig. 3g and Supplementary Fig. S3i). The inhibition effect of CQ is comparable to a furin inhibitor decanoyl-RVKR-chloromethylketone (CMK) (Supplementary Fig. S3j). Since CQ inhibits lysosomal acidification, we concluded that the selective cleavage of spike protein is lysosome-dependent but not autophagosome-dependent.

### SARS-CoV-2 is endocytosed by clathrin-mediated endocytosis

**Fig. 4:**
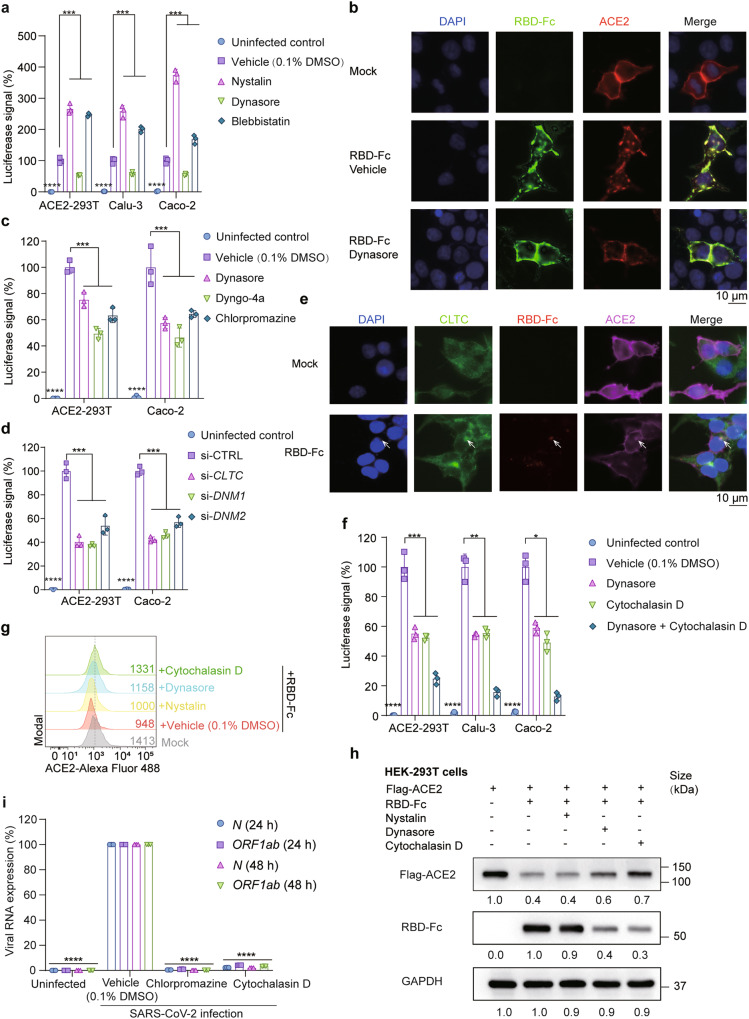
ACE2 is endocytosed by clathrin-mediated endocytosis. **a** Luciferase signals showing SARS-CoV-2 pseudovirus entry into three cell lines (ACE2-293T, Calu-3, Caco-2) with different endocytosis inhibitors (nystatin (10 μM), caveolae-mediated endocytosis inhibitor; dynasore (20 μM), clathrin-mediated endocytosis inhibitor; blebbistatin (20 μM), macropinocytosis inhibitor) or vehicle (0.1% DMSO) at 48 h post-infection. Viral entry efficiency was measured as the percentages of luciferase signal with inhibitors/vehicle (0.1% DMSO) treated cells. Error bars indicate SEM (*n* = 3). *P*-values were calculated by Tukey’s multiple comparison test. ***P < 0.001; ****P < 0.0001. **b** Immunofluorescent microscopy images showing the localization of ACE2 (red) in Flag-ACE2 transfected HEK293T cells treated with or without RBD-Fc (green) or dynasore (20 μM). Cell nuclei were stained with DAPI (Blue). Colocalization of ACE2 and RBD-Fc was indicated by white arrows. Scale bars, 10 μm. **c** Luciferase signals showing entry of SARS-CoV-2 pseudovirus into ACE2-293T and Caco-2 with three clathrin-mediated endocytosis inhibitors (dynasore (20 μM), dyngo-4a (20 μM), chlorpromazine (25 μM)) or vehicle (0.1% DMSO). Viral entry efficiency was measured as the percentages of luciferase signal with inhibitors/vehicle (0.1% DMSO) treated cells. Error bars indicate SEM (*n* = 3). *P*-values were calculated by Tukey’s multiple comparison test. ***P < 0.001; ****P < 0.0001. **d** Luciferase signals showing entry of SARS-CoV-2 pseudovirus into ACE2-293T and Caco-2 with siRNA targeting *CLTC*, *DNM1*, *DNM2* or a control RNA. Viral entry efficiency was measured as the percentage of luciferase signal with siRNA targeting a specific gene/control siRNA (si-CTRL). Error bars indicate SEM (*n* = 3). *P*-values were calculated by Tukey’s multiple comparison test. ***P < 0.001; ****P < 0.0001. **e** Immunofluorescent microscopy images showing colocalization of ACE2 (magenta), CLTC (green) and RBD-Fc protein (red) in Flag-ACE2 transfected HEK-293T cells at 3 h post pseudovirus infection. Cell nuclei were stained with DAPI (Blue). Scale bars, 10 μm. Colocalization of CLTC with ACE2 and RBD-Fc protein is indicated by white arrows. **f** Entry of SARS-CoV-2 pseudovirus into three cell lines (ACE2-293T, Calu-3, Caco-2) with vehicle (0.1% DMSO), dynasore (20 μM) or/and cytochalasin D (10 μM). Viral entry efficiency was measured as the percentage of luciferase signal with inhibitors/vehicle (0.1% DMSO) treated cells. Error bars indicate SEM (*n* = 3). *P*-values were calculated by Tukey’s multiple comparison test. ***P < 0.001; ****P < 0.0001. **g** Histograms of ACE2 surface expression in ACE2-HEK-293T cells treated with RBD-Fc in the presence of vehicle (0.1% DMSO), dynasore (20 μM), cytochalasin D (10 μM) or nystatin (10 μM). **h** Immunoblot analysis showing extracts of Flag-ACE2 transfected 293T cells treated with RBD-Fc, nystatin (10 μM), dynasore (20 μM) and/or cytochalasin D (10 μM) for 6 h. **i** Relative viral RNA (*N* and *ORF1ab*) expression level in addition to chlorpromazine (25 μM) (clathrin-mediated endocytosis inhibitor) or cytochalasin D (10 μM) (cytoskeleton remodeling inhibitor) at 24 and 48 h after SARS-CoV-2 infection. RNA expression levels were normalized to vehicle (0.1% DMSO) at indicated time points. *P*-values were calculated by Tukey’s multiple comparison test. ****P < 0.0001. Unless otherwise specified, *n* = 3 biologically independent experiments were performed (**a**–**i**)

Endocytic processes include clathrin-mediated endocytosis, caveolin-mediated endocytosis and macropinocytosis. To determine how SARS-CoV-2 enters the host cell, we tested dynasore (a dynamin inhibitor that suppresses clathrin-mediated endocytosis), nystatin (an inhibitor that suppressescaveolae-mediated endocytosis) and blebbistatin (a selective myosin II ATPase inhibitor that inhibits macropinocytosis). The inhibitors were applied in non-toxic concentrations determined in Supplementary Fig. S4a. Dynasore reduced luciferase activity by about 50% (Fig. 4a), supporting clathrin-dependent endocytosis. In contrast, nystatin and blebbistatin increased pseudovirus uptake in all cell lines. A possible explanation is that blocking caveolin-mediated endocytosis and macropinocytosis augments clathrin-mediated endocytosis. Consistently, endocytosis of RBD-Fc and ACE2 was prohibited by dynasore (Fig. 4b). We further assessed pseudovirus entry with other clathrin-mediated endocytosis inhibitors (dyngo-4a, dynamin inhibitor; chlorpromazine, inhibitor of the assembly and disassembly of clathrin lattices on cell surfaces). These inhibitors also blocked the entry of pseudovirus with 50% efficacy (Fig. 4c). Clathrin heavy chain (CLTC), Dynamin 1 (DMN1) and Dynamin 2 (DMN2) are genes responsible for clathrin-mediated endocytosis. Knockdown with siRNAs targeting these genes led to significant decreases of luciferase signals upon pseudovirus infection (Fig. 4d). The efficiencies of these siRNAs were assayed in Supplementary Fig. S4b. In response to RBD-Fc treatment, CLTC clustered and colocalized with RBD-Fc (Fig. 4e and Supplementary Fig. S4c).

Since viral entry by endocytosis requires cytoskeleton rearrangement, we evaluated viral entry efficiency with the treatment of cytochalasin D, a chemical compound that inhibits actin polymerization. Cytochalasin D inhibited pseudovirus entry by around 50%. The combination of cytochalasin D and dynasore further limited the uptake of pseudoviruses to around 10–20% (Fig. 4f). When using cytochalasin D and dynasore, the surface expression of ACE2 was retained, as confirmed by flow cytometry (Fig. 4g). Both treatments inhibited RBD-Fc uptake and restored ACE2 expression (Fig. 4h and Supplementary Fig. S4d). Consistent with findings using pseudovirus, we also found that chlorpromazine and cytochalasin D inhibited the propagation of viral RNA (N and Orf1ab) upon SARS-CoV-2 infection (Fig. 4i). Taken together, we show that clathrin-mediated endocytosis and cytoskeleton rearrangement mediate SARS-CoV-2 entry into host cells.

### PAK1-mediated cytoskeleton rearrangement is responsible for viral entry

**Fig. 5:**
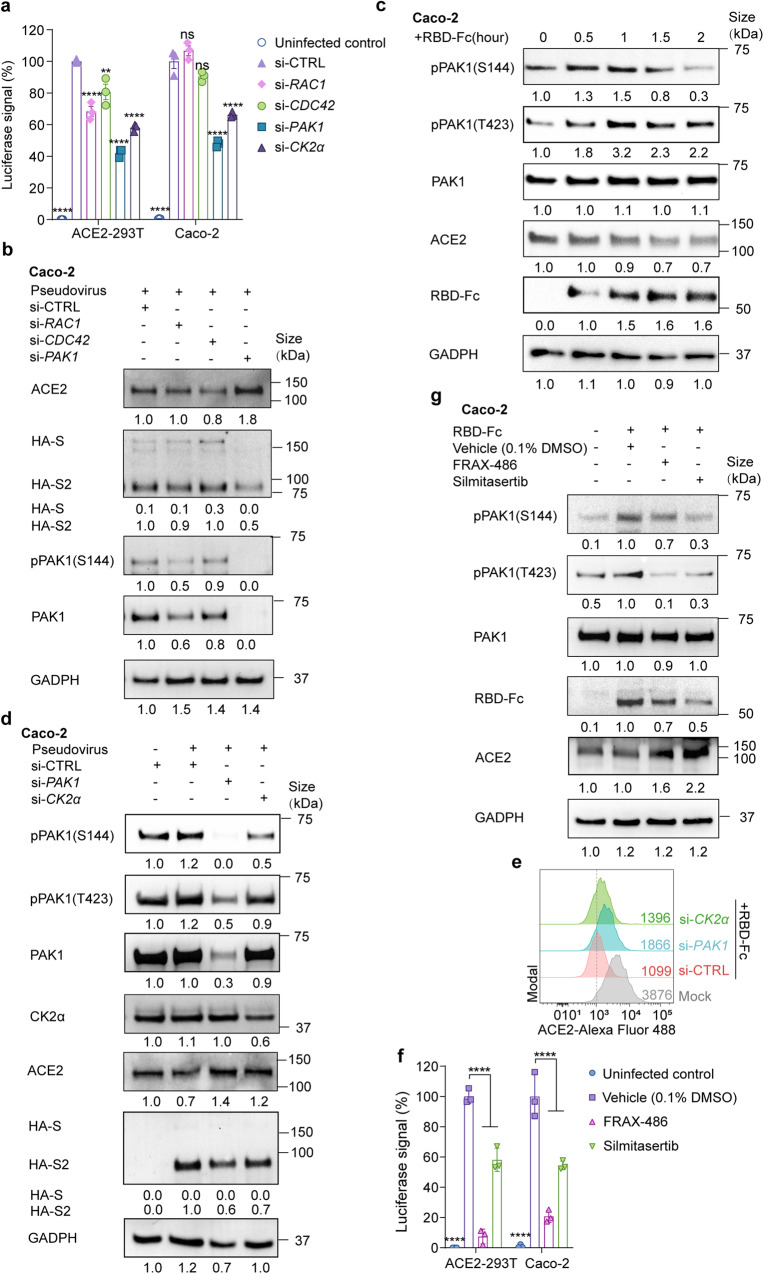
CK2 and PAK1 mediate endocytosis of ACE2 and viral particles. **a** Entry of SARS-CoV-2 pseudovirus into ACE2-293T and Caco-2 cells transfected with siRNA targeting *RAC1, CDC42, PAK1, CK2α* or a control siRNA (si-CTRL). Viral entry efficiency was measured as the percentage of luciferase signal with siRNA targeting a specific gene/control siRNA (si-CTRL) transfected cell. Error bars indicate SEM (*n* = 3). *P*-values were calculated by Tukey’s multiple comparison test. ****P < 0.001; ns: not significant. **b** Immunoblot analysis of extracts of Caco-2 cells transfected with siRNA targeting *RAC1, CDC42, PAK1* or a control siRNA (si-CTRL) and infected with pseudovirus. S and S2 were detected by an anti-HA antibody. **c** Immunoblot analysis of extracts of Caco-2 cells with the incubation of RBD-Fc at indicated time points. **d** Immunoblot analysis of extracts of Caco-2 cells transfected with a control siRNA (si-CTRL), *PAK1*, or *CK2α*, and infected with pseudovirus. S and S2 were detected by an anti-HA antibody. **e** Histograms of ACE2 surface expression in ACE2-HEK-293T cells transfected with a control siRNA (si-CTRL), *CLHC*, or *PAK1*. **f** Entry of SARS-CoV-2 pseudovirus into ACE2-293T and Caco-2 cells with FRAX-486 (2.5 μM), silmitasertib (2.5 μM) or vehicle (0.1% DMSO). Viral entry efficiency was calculated as the percentage of luciferase signal with inhibitors/vehicle (0.1% DMSO) treated cells. Error bars indicate SEM (*n* = 3). *P*-values were calculated by Tukey’s multiple comparison test. ***P < 0.001. **g** Immunoblot analysis of extracts of RBD-Fc treated Caco-2 cells incubated with or vehicle (0.1% DMSO), FRAX-486 (2.5 μM) or silmitasertib (2.5 μM). Unless otherwise specified, *n* = 3 biologically independent experiments were performed (**a**–**g**)

PAK1 is a specific activator for cytoskeleton remodeling that enables the endocytosis of vaccinia virus,^37^ African swine fever virus,^38^ and respiratory syncytial virus.^39^ We found that PAK1 was also required for SARS-CoV-2 endocytosis and infectivity (Fig. 5a). Knockdown of PAK1 restored ACE2 level and inhibited RBC-Fc uptake (Fig. 5b). The efficiency of the siRNA was confirmed in Supplementary Fig. S5a. In a kinetic study using RBD-Fc treated Caco-2 cells, we further confirmed PAK1 activation by measuring phosphorylation of Ser144 and Thr423 (Fig. 5c and Supplementary Fig. S5b).

PAK1 activation relies on EGFR and Rho family GTPases (RAC1/CDC42)^38^ (Supplementary Fig. S5c). Here, we found that silencing RAC1 or CDC42 did not prevent pseudovirus infectivity nor restore ACE2 expression. In addition, genistein, a protein tyrosine kinase (PTK) inhibitor that blocks EGFR signaling, did not prevent viral endocytosis (Supplementary Fig. S5d), suggesting that PAK1 was not activated by the classical EGFR/RAC1/CDC42 pathway.

CK2α promotes RAC1/CDC42-independent PAK1 activation.^40^ It has been reported recently that CK2α was activated in SARS-CoV-2 infection.^41^ We found that knocking down CK2α decreased PAK1 activation (Fig. 5d) and restored surface ACE2 level (Fig. 5e). Finally, we utilized the pan-PAK inhibitor FRAX-486 and the CK-2α inhibitor silmitasertib and found that they both significantly decreased pseudovirus entry (Fig. 5f) as well as RBD-Fc mediated ACE2 degradation (Fig. 5g). Similar results were also observed using Calu-3 cells (Supplementary Fig. S5e). The non-toxic concentration of these inhibitors was pre-determined (Supplementary Fig. S5f).

### FRAX-486 exhibits in vitro and in vivo anti-SARS-CoV-2 activity

**Fig. 6:**
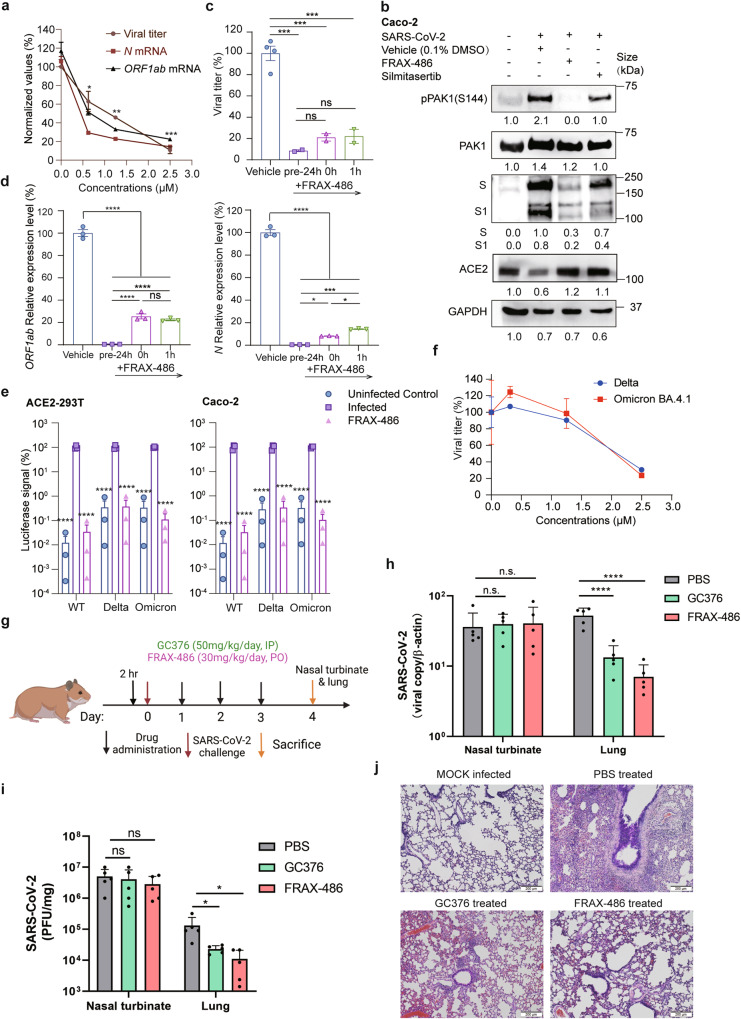
FRAX-486 inhibits SARS-CoV-2 infections in vitro and in vivo. **a** Concentration-dependent inhibition of viral titer and viral RNA (N and *ORF1ab*) expression upon the treatment of FRAX-486 in SARS-CoV-2-infected Caco-2 cells (MOI = 0.01) at 48 h post-infection. Values were normalized as the percentage of infected cells treated with inhibitors/untreated (0 μM). Error bars indicate SEM (*n* = 2 for viral titer, *n* = 3 for viral RNA). *P*-values were calculated by comparing the parentage of cells treated with inhibitors with untreated infected cells by Tukey’s multiple comparison test. *P < 0.05, **P < 0.01, ***P < 0.001. **b** Immunoblot analysis of extracts of Caco-2 cells infected by SARS-CoV-2 with the treatment of vehicle (0.1% DMSO), FRAX-486 (2.5 μM) or silmitasertib (2.5 μM) (MOI = 0.5) at 3 h post-infection. **c** Inhibition of viral titer pre- and post-treatment with FRAX-486 (2.5 μM) or vehicle (0.1% DMSO) in SARS-CoV-2-infected Caco-2 cells in indicated time points (MOI = 0.01). Error bars indicate SEM (*n* = 2). *P*-values were calculated by Tukey’s multiple comparison test. *P < 0.05, ***P < 0.001, ****P < 0.0001, ns: not significant. **d** Inhibition of viral RNA (N and *ORF1ab*) pre- and post-treatment with FRAX-486 (2.5 μM) or vehicle (0.1% DMSO) in SARS-CoV-2-infected Caco-2 cells in indicated time points (MOI = 0.01). Error bars indicate SEM (*n* = 3). *P*-values were calculated by Tukey’s multiple comparison test. *P < 0.05, ***P < 0.001, ****P < 0.0001, ns: not significant. **e** Entry of SARS-CoV-2 pseudovirus variants (wild-type, delta and omicron) into ACE2-293T and Caco-2 cells with FRAX-486 (2.5 μM) or vehicle (0.1% DMSO). Viral entry efficiency was calculated as the percentage of luciferase signal with inhibitors/vehicle (0.1% DMSO) treated cells. Error bars indicate SEM (*n* = 3). *P*-values were calculated by Tukey’s multiple comparison test. ***P < 0.001. **f** Inhibition of viral titer upon the treatment of FRAX-486 in A549 cells infected by SARS-CoV-2 variants (delta and omicron BA4.1) at 48 h post-infection. **g** Schematic of the hamster model for evaluation of the in vivo anti-SARS-CoV-2 effect of FRAX-486. **h** SARS-CoV-2 RNA loads in SARS-CoV-2-infected Syrian hamsters treated with FRAX-486, GC376 (positive control) or PBS (negative control) (*n* = 5). **i** SARS-CoV-2 virus titers determined by plaque assay in SARS-CoV-2-infected Syrian hamsters treated with FRAX-486, GC376 (positive control) or PBS (*n* = 5). **j** Hematoxylin and eosin staining of SARS-CoV-2-infected hamster lungs treated with FRAX-486, GC376 (positive control) or PBS (negative control) (*n* = 5). Scale bars, 10 μm

The in vitro antiviral activity of FRAX-486 and silmertasertib were compared. FRAX-486 showed a higher inhibition activity in viral RNA replication than silmertasertib (Supplementary Fig. S6). We further examined the antiviral effects of FRAX-486 using live SARS-CoV-2 in vitro and in vivo. FRAX-486 displayed dose-dependent inhibitory effects on both viral titers and viral RNA N and ORF1ab genome copies (Fig. 6a) with an IC_50_ of approximately 1.25 μM and a selective index of 8 in SARS-CoV-2-infected Caco-2 cells. In vitro treatment with FRAX-486 or silmitasertib reduced viral replication by inhibiting PAK1 phosphorylation (Fig. 6b). In a time-of-drug-addition assay, FRAX-486-treated cells at all stages exhibited reduced SARS-CoV-2 replication and expression of viral proteins (Fig. 6c, d). FRAX-486 prevented the entry of pseudotyped SARS-CoV-2 variants delta and omicron (Fig. 6e) and inhibited the proliferation of SARS-CoV-2 variants delta and omicron (Fig. 6f). In conclusion, we have validated the broad-spectrum anti-SARS-CoV-2 activity of FRAX-486 in vitro.

The Syrian hamster model has been shown as a reliable tool for assessing transmission, pathogenesis and therapeutics against SARS-CoV-2.^42^ We assessed the in vivo antiviral activity of FRAX-486 in our study using this model (Fig. 6g). We showed that FRAX-486 exhibited potent anti-SARS-CoV-2 effect that was comparable to that of the positive control 3CL protease inhibitor GC376^11^ (Fig. 6h–j). FRAX-486-treated hamsters had significantly decreased lung viral RNA load and infectious virus titers (Fig. 6h, i). Histopathological examination revealed less severe pulmonary inflammatory infiltrates and hemorrhage compared to phosphate-buffered saline (PBS)-treated control hamsters (Fig. 6j). Taken together, we identify that the pan-PAK inhibitor FRAX-486 is a novel host-targeting candidate drug for COVID-19.

## Discussion

**Fig. 7:**
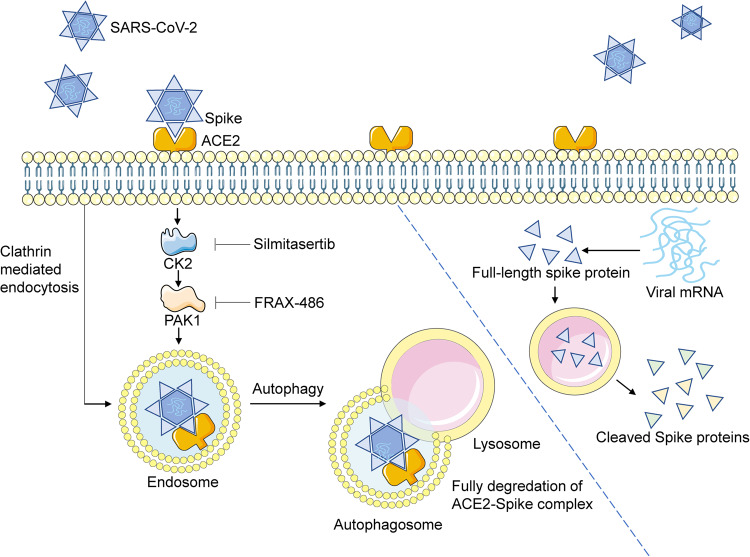
ACE2-spike complex is degraded by clathrin-mediated endocytosis and PAK1-mediated cytoskeleton rearrangement. Full-length spike protein without ACE2 binding is selectively cleaved into S1 and S2 subunits in a lysosomal-dependent manner. Viral entry can be inhibited by CK2 or PAK1 inhibitors

Although it is well known that ACE2 is the receptor for SARS-CoV-2, how it mediates viral entry and how it is degraded during this process have not been fully elucidated. Fusion and endocytosis are the two major pathways of SARS-CoV-2 cell entry.^43^ In this study, we show that SARS-CoV-2 spike protein promotes ACE2 endocytosis by clathrin- and PAK1-mediated cytoskeleton rearrangement. At the stage of viral entry, following endocytosis, the ACE2-spike protein complex is degraded in an autophagic-dependent manner. This facilitates the release of the viral genome. At the stage of viral replication and packaging, spike proteins exist as monomers and are selectively cleaved into S1 and S2 subunits. ACE2 inhibits selective cleavage of the viral spike proteins and therefore may decrease viral infectivity.^22^ We show that PAK1 inhibition restores ACE2 expression and, most importantly, decreases viral loads and lung pathology in vivo (Fig. 7).

Restoring ACE2 surface expression may ameliorate disease severity by suppressing the overactivation of the renin angiotensin system (RAS).^24^ ACE2 expression prevents lung injury caused by various respiratory viruses, such as SARS-CoV, avian influenza, and respiratory syncytial virus.^25,44–46^ Elderly patients with cardiovascular disease, diabetes, and/or hypertension are at increased risk of developing severe or fatal COVID-19.^47,48^ ACE2 downregulation has been found in these diseases.^49*–*52^ We showed that SARS-CoV-2 spike protein hijacks and degrades ACE2 by autophagy during infection, which leads to depletion of membrane- and intracellular-localized ACE2. ACE2 deficiency of the infected cells may facilitate the virus to infect bystander cells that have not been previously infected, therefore improving the overall efficiency for viral spread. On the other hand, ACE2 inhibits the selective cleavage of viral spike protein and promotes its degradation, which may restrict viral propagation in the replication stage. Infection-mediated ACE2 degradation raises the levels of AngII. Plasma AngII levels in COVID-19 patients are markedly elevated and positively correlated with viral titers.^53^ Excessive AngII increases lung hydrostatic pressure and microvascular permeability, resulting in inflammatory cell infiltration, pulmonary edema and fibroblast proliferation.^54*–*56^ These findings suggest that ACE2 also plays a protective role in COVID-19. It should be noted that ADAM17 has been shown to mediate cleavage and shedding of ACE2 during SARS-CoV infection.^26*–*28^ In our study, we found that ADAM17 did not mediate ACE2 shedding during SARS-CoV-2 infection. ACE2 was not detected in the supernatant of SARS-CoV-2 pseudovirus-infected cells; the application of ADAM17 inhibitor TAPI-1 did not restore ACE2 expression. This conclusion has certain limitations because SARS-CoV was not included as a positive experimental control for ADAM17-mediated ACE2 shedding.

By using the SARS-CoV-2-infected hamster model in vivo, we showed that the pan-PAK inhibitor FRAX-486 effectively suppressed viral propagation in the lung tissue. Intriguingly, FRAX-486 did not suppress SARS-CoV-2 propagation in the hamster’s nasal turbinate. Recently, Wu et al. showed that PAK1 and PAK4 were activated in SARS-CoV-2-infected human nasal epithelial cells and that FRAX-486 did not suppress viral entry but instead inhibited a late step in viral replication in vitro.^57^ The differences in the requirement of PAK1 for viral entry between Wu’s and our study may reflect host cell origin and experimental settings. Although both we and Wu showed that PAK1 was activated in response to SARS-CoV-2 infection, PAK1 activation occurred 2 h post-infection in Caco-2 cells in our study, while PAK1 activation occurred 36 h post-infection in the human nasal epithelial cells in Wu’s study. Since PAK1 was not activated in the early viral entry stage in nasal epithelial cells, FRAX-486 would not be expected to have an effect on viral entry 24 h post-infection, as shown in Wu’s experiments.^57^ Moreover, published studies have shown that human neutralizing antibodies or DNA vaccination significantly reduced infection in the lungs but not in the nasal turbinate of hamsters intranasally challenged with SARS-CoV-2.^58^ Nasal epithelium cells express high levels of ACE2 and TMPRSS2, and tends to display inactive antiviral innate immune responses than lung epithelial cells when challenged with SARS-CoV-2.^59,60^ Future studies, particular in vivo studies are required to determine if PAK1 inhibitors may prevent viral propagation in nasal epithelial cells.

A major obstacle to developing antiviral therapeutics is the continuous emergence of new viral strains. Viral proteins, such as main protease^61^ and RNA-dependent RNA polymerase,^62^ are the major therapeutic targets of SARS-CoV-2. The SARS-CoV-2 RNA polymerase inhibitor remdesivir, 3CL protease inhibitor nirmatrelvir/ritonavir, and the RNA synthesis inhibitor azvudine have been approved for the treatment of COVID-19 patients by FDA or CFDA.^62*–*65^ GC373, GC376, boceprevir,^66,67^ ebselen^68^ and a set of chemicals with the common structure of α-ketoamides^61^ inhibit 3CLpro protease activity in low to median micromolar ranges in vitro. The main concern for viral protein inhibitors is the emergence of drug-resistant mutants. Likewise, the protective effects of vaccines are decreased for the Omicron variants.^69^ We showed that the pan-PAK inhibitor FRAX-486 is a potent host-targeting anti-SARS-CoV-2 compound that prevents WT, delta and omicron entry in vitro. FRAX-486 also achieved comparable antiviral effects compared with the 3CLpro protease GC376 in vivo in the hamster model. Further evaluation of FRAX-486 in clinical trials for COVID-19 should be considered.

In summary, we show that PAK1 is an attractive host factor that may be targeted for the therapeutic purpose of COVID-19. We envisage that PAK1 inhibition may alleviate disease severity by inhibiting viral propagation and restoring ACE2 expression.

## Material and methods

### Biosafety and ethics approval

All works with live SARS-CoV-2 were conducted in the Biosafety Level 3 (BSL3) Laboratories of Guangzhou Customs District Technology Center or the University of Hong Kong. The animal experiments in this study were approved by the HKU Committee on the Use of Live Animals in Teaching and Research.

### Viruses and cell lines

The SARS-CoV-2 strains used in this study were SARS-CoV-2/human/CHN/IQTC01/2020 (GenBank accession no. MT123290.1), SARS-CoV-2/B.1.617.2/Delta (GenBank: OM212471) and SARS-CoV-2/ Hong Kong/HKU-220712-004/2022 (GISAID accession no. EPI_ISL_13777657). SARS-CoV-2/human/HKG/HKU-001a/2020 (GenBank accession no. MT230904) were used for in vivo studies. The following cell types were used in this study: HEK293T (ATCC CRL-3216), ACE2-293T (HEK293T cells stably expressing recombinant human ACE2), Calu3 (ATCC HTB-55), and Caco-2 (ATCC HTB-37). HEK293T, ACE2-293T and Calu3 were maintained in Dulbecco’s modified Eagle’s medium supplemented with 10% fetal calf serum.^70,71^ Caco-2 were kept in minimum essential medium supplemented with 20% fetal calf serum.

### Biological and chemical materials

The coding DNA sequences of ACE2 (GenBank accession: AB046569.1) and full-length spike protein (GenBank accession: QHD43416.1) were cloned to pENTER-flag and pcDNA3.1. The spike protein RBD domain (aa. 319-541) fused with a mouse Ig tag (Cat# BTMY002) and his-tagged full-length spike protein (Cat# BTMY003) were commercial products purchased from Biotube Biotechnology Co., Ltd.

The following antibodies were used for western blot, immunofluorescence and flow cytometry: anti-human ACE2 antibody (CST, Cat#4355S), HRP conjugated anti-Flag antibody (Sigma-Aldrich, Cat#A8592), HRP conjugated anti-Myc antibody (CST, Cat# 2040S), anti-His antibody (CST, Cat#D3I1O), HRP conjugated anti-HA (CST, Cat# 2999S), anti-human GADPH antibody(CST, Cat# D4C6R), HRP conjugated anti-Rabbit IgG (CST, Cat#7074S), HRP conjugated anti-Mouse IgG (CST, Cat#7076S), anti-LC3B (CST, Cat#3868), anti-human pPAK1(S144) (CST, Cat#2606), anti-pPAK1(T423) (CST, Cat#2606), anti-human PAK1(CST, Cat#2602), anti-CHLC(Abcam, Cat#ab2731) (CST, Cat #4796), anti-CK2α (CST, Cat #2656S), anti-SARS-CoV-2 spike protein (SinoBiological, Cat#40591-MM42).

The working concentration and resource of chemicals used in this study were listed in Supplementary Table 1. The working concentration of these chemicals was either determined by CCK8 cell viability assay before use or by previous reports.

### Coronavirus spike-mediated pseudovirus entry assay

Coronavirus spike-mediated pseudovirus was generated as previously described.^33^ To determine the infectivity of pseudovirus, virus concentrations following a 10-fold serial dilution were used to infect ACE2-293T cells. At 3 h post-infection, flow cytometry was performed to monitor cell infectivity. The minimum viral concentration that resulted in nearly 100% of GFP-positive cells was considered as MOI = 1. For the assay of viral entry, cells were infected at MOI = 1. After 48 h, cells were washed and lysed for detection of luciferase signal using Promega Luc steady glo.

### Cell viability test

To determine the cell viability with chemicals by Cell counting kit-8 (CCK8), cells grown to 50% confluency in 96-well plates were applied with chemicals with 2-fold diluted concentrations. Wells with 0.1% DMSO were included as control. At 48 h post-incubation, 10 μl of CCK8 solution was added to the well and incubated for 3 h. The absorbance of 450 nm of the plate was measured by Thermo Scientific Multiskan GO. Results are shown in Supplementary Figs. S1f, S3h, S4a and S5f.

### Immunoblot analysis

Cells were lysed in 100 μl low salt lysis buffer (150 mM NaCl, 1% Triton-X100, 0.25 mM HEPES, 3.2 mM MgCl_2_, 1 mM EDTA, 10% glycerol) on ice for 1 h. Then, 20 μl of 5X SDS loading dye was added to cell lysates and boiled at 95 °C for 10 min. The proteins were resolved to SDS-PAGE and transferred to PVDF membranes. Appropriate antibodies were used to detect target proteins. Immobilon Western Chemiluminescent HRP Substrate (Millipore) was used for the image blot. Images were processed using Image Lab 3.0, Bio-Rad.

### Immunofluorescence assay

Cells were fixed with 4% paraformaldehyde on ice for 15 min. Then cells were permeabilized in methanol in −20 °C for 30 min. Then cells were washed in blocking buffer (2% BSA in PBS) for 15 min, 3 times and stained in primary antibody at 4 °C overnight. On the next day, cells were washed (blocking buffer for 15 min, 3 times), followed by secondary antibody incubation for 1 h at room temperature in the dark. Then cells were washed and stained in VECTASHIELD Antifade Mounting Medium with DAPI (Vector Laboratories, Cat#H-1200) for 5 min and washed in a blocking buffer. Immunofluorescent images were obtained with a Leica TCS SP8 Inverted Fluorescence Microscope (Leica Microsystems) and analyzed with Leica X image analysis software (Leica) and Image J.^72^

### Flow cytometry

Cells were trypsinized and then resuspended in FACS buffer (PBS with 2% FBS and 1 mM EDTA). Cells were stained with a primary antibody for 1 h on ice. After wash, cells were stained with a secondary antibody for 1 h at room temperature. Data were acquired using a BD FACS Aria Sorp and analyzed by Flowjo 10.4 software (FlowJo LLC).

### Transfections for plasmids and siRNA

Plasmids were transfected to cells using Lipofectamine3000 (Thermofisher, Cat# L3000015) according to the manufacturer’s instructions. SiRNA was transfected by Lipofectamine RNAiMax (Thermofisher, Cat#13778150). The sequences of siRNA are listed in Supplementary Table 2. RNA silencing efficiency was validated by real-time PCR, as shown in Supplementary Figs. S3e, S4b and S5a.

### Real-time PCR

An EZ-press RNA purification Kit (EZbiosciences, Cat#B0004DP) was used to isolate total RNA. Here, 1 μg RNA was reverse transcribed by in the HiScript III 1st Strand cDNA Synthesis Kit (Vazyme, Cat# R312-01). Real-time PCR was performed with ChamQ Universal SYBR qPCR Master Mix (Vazyme, Cat# Q711-02). Data were recorded using Applied Biosystems Quantstudio 6 Flex. All procedures followed the standard instructions according to the manufacturer. Primers were listed in Supplementary Table 3. Each PCR reaction was performed in triplicates, and the mean Ct value was used for statistical analysis. Messenger RNA expression was standardized to GADPH expression levels, followed by normalization to the control group.

### Plaque assay

Virus or lung homogenate supernatants were serially diluted in DMEM. After viral inoculation, plates were overlaid with 1.2% agarose containing 4% FBS. At 48 h post-infection, the agar was removed, and cells were stained with 0.1% crystal violet. Viral titers were calculated as plaque-forming unit (PFU) per ml or per gram tissue.

### Animal model

Male Syrian hamsters, aged 8–10 weeks, were obtained from the Chinese University of Hong Kong Laboratory Animal Service Centre through the University of Hong Kong Centre for Comparative Medicine Research. The animals were kept in BSL-2 housing and given access to standard pellet feed and water ad libitum.^73^ The hamsters were randomly assigned to receive oral FRAX-486 (30 mg/kg/day), intraperitoneal GC376 (50 mg/kg/day), or intraperitoneal PBS starting at 6 h before the virus challenge and continued daily for a total of four doses. On day 0 post-infection (dpi), each hamster was intranasally challenged with 50 μl of 2 × 10^6^ plaque-forming units/ml of SARS-CoV-2 as previously described.^42^ An additional group of mock-infected hamsters were included as controls. Five animals per group were sacrificed at 4 dpi for virological and histopathological assessment as previously described.^74^

### Statistics

All of the data were analyzed using GraphPad Prism version 9.0 (GraphPad Software, Inc., San Diego, CA, USA). Unpaired t-test was used for two groups’ analysis and a one-way ANOVA analysis of variance for three or more groups’ analysis. *P*-values were adjusted by Tukey’s multiple comparison test or Dunnett’s multiple comparisons test.

### Supplementary information

Supplementary Materials

Data S1. All original films of Western blots

## Data Availability

Data supporting the results of the present work are available in this manuscript and supplementary materials and also from the corresponding authors upon reasonable request.
