# Supplementary material for: P21-activated kinase 1 (PAK1)-mediated cytoskeleton rearrangement promotes SARS-CoV-2 entry and ACE2 autophagic degradation
Source: Signal Transduct Target Ther. 2023 Oct 9;8:385. doi: 10.1038/s41392-023-01631-0 (PMC10560660; doi:10.1038/s41392-023-01631-0)
Supplement: Supplementary file 1 — Supplementary Materials [file 41392_2023_1631_MOESM1_ESM.docx]

Supplementary Materials for

P21-activated kinase 1 (PAK1)-mediated cytoskeleton rearrangement promotes SARS-CoV-2 entry and ACE2 autophagic degradation

Ming Liu, Bingtai Lu, Yue Li, Shuofeng Yuan, Zhen Zhuang, Guangyu Li, Dong Wang, Liuheyi Ma, Jianheng Zhu, Jinglu Zhao, Chris Chung-Sing Chan, Vincent Kwok-Man Poon, Kenn Ka-Heng Chik, Zhiyao Zhao, Huifang Xian, Jingxian Zhao, Jincun Zhao, Jasper Fuk-Woo Chan, Yuxia Zhang

Correspondence to: Yuxia Zhang [yuxia.zhang@gwcmc.org](file:///D:\Downloads\%20mailto:yuxia.zhang@gwcmc.org), Jasper Fuk-Woo Chan [jfwchan@hku.hk](file:///D:\Downloads\%20mailto:jfwchan@hku.hk) and Jincun Zhao [Zhaojincun@gird.cn](mailto:Zhaojincun@gird.cn)

**This PDF file includes:**

Supplementary Figure legends

Figures S1 to S6

Tables S1 to S3

References

**Other Supplementary Materials for this manuscript include the following:**

Data S1


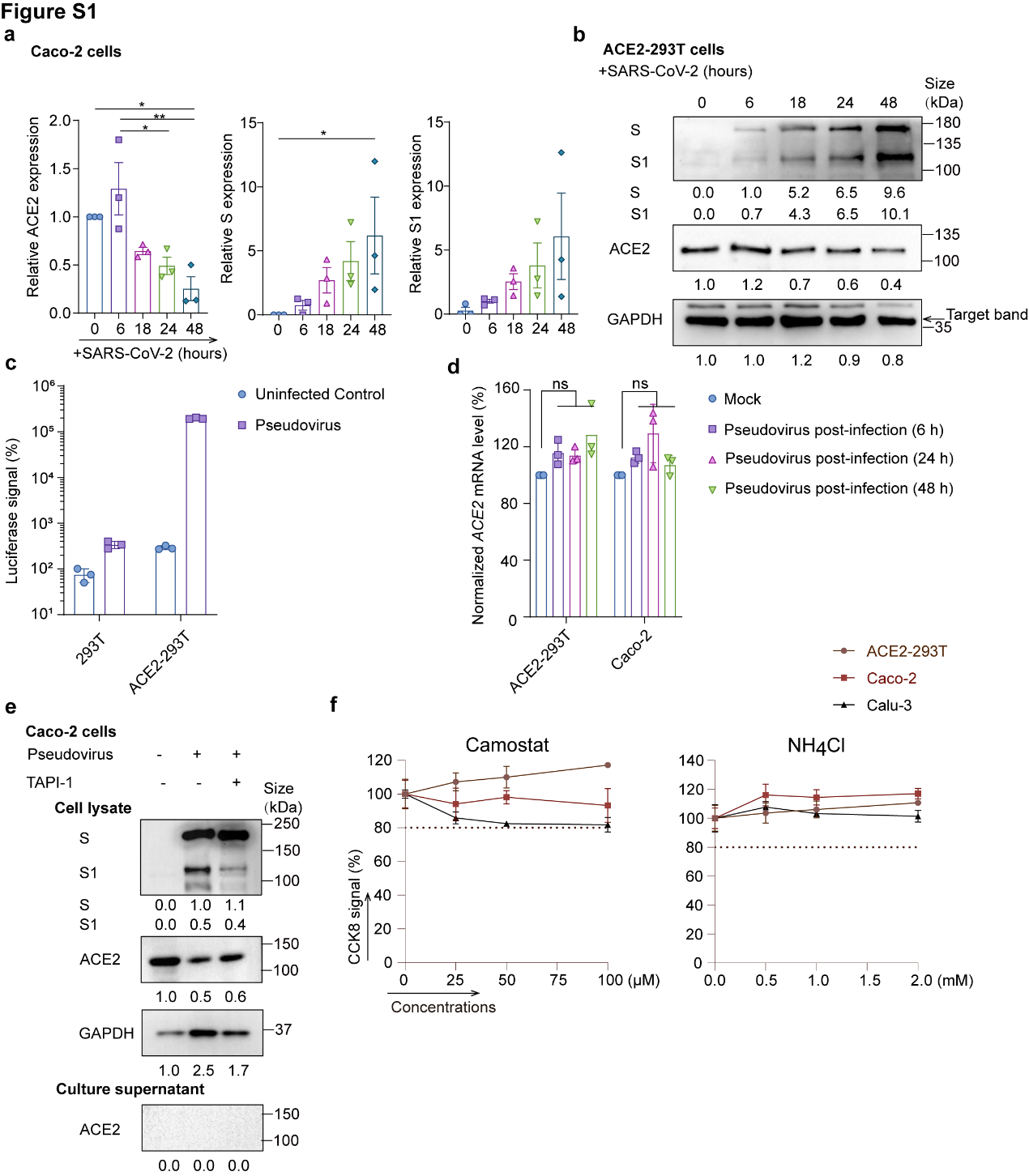


Figure. S1. Packaged SARS-CoV-2 pseudovirus endorses ACE2 degradation in protein level.

1. Statistic diagrams showing relative protein levels of ACE2, S and S1 in **Figure 1a**. Error bars indicate SEM. *P*-values were calculated by Tukey’s multiple comparison test. * *P* < 0.05, ** *P* < 0.01. All unlabeled comparisons are not significant.
2. Immunoblot analysis of extracts of ACE2-293T cells infected by SARS-CoV-2 at 6, 18, 24, 48 hours post-infection (MOI=0.01). S and S1 were detected using an anti-S1 antibody.
3. Luciferase signal showing SARS-CoV-2 pseudovirus entry of 293T and ACE2-293T cells at 48 hours post-infection.
4. ACE2 mRNA level during pseudovirus infection at time points. Caco-2 cells were infected by wild-type SARS-CoV-2 pseudovirus for 6, 24 and 48 hours. mRNA level was detected by qPCR. Error bars indicate SEM. ns: not significant.
5. Immunoblot analysis of extracts of Caco-2 cells and cell culture supernatants infected by SARS-CoV-2 pseudovirus for 6 hours with/without ADAM17 inhibitor TAPI-1 (1 μM).
6. Concentration dependent chemical cytotoxicity of Camostat and NH_4_Cl measured by Cell Counting Kit-8 (CCK-8). Cells were treated with the chemicals at indicated concentration for 48 hours. CCK8 signals were normalized to the signal at 0 μM. Error bars indicate SEM (n=4). * *P* < 0.05, ** *P* < 0.01, *** *P* < 0.001, **** *P* < 0.0001. Unless otherwise specified, *n*=3 biologically independent experiments were performed (**a-f**).


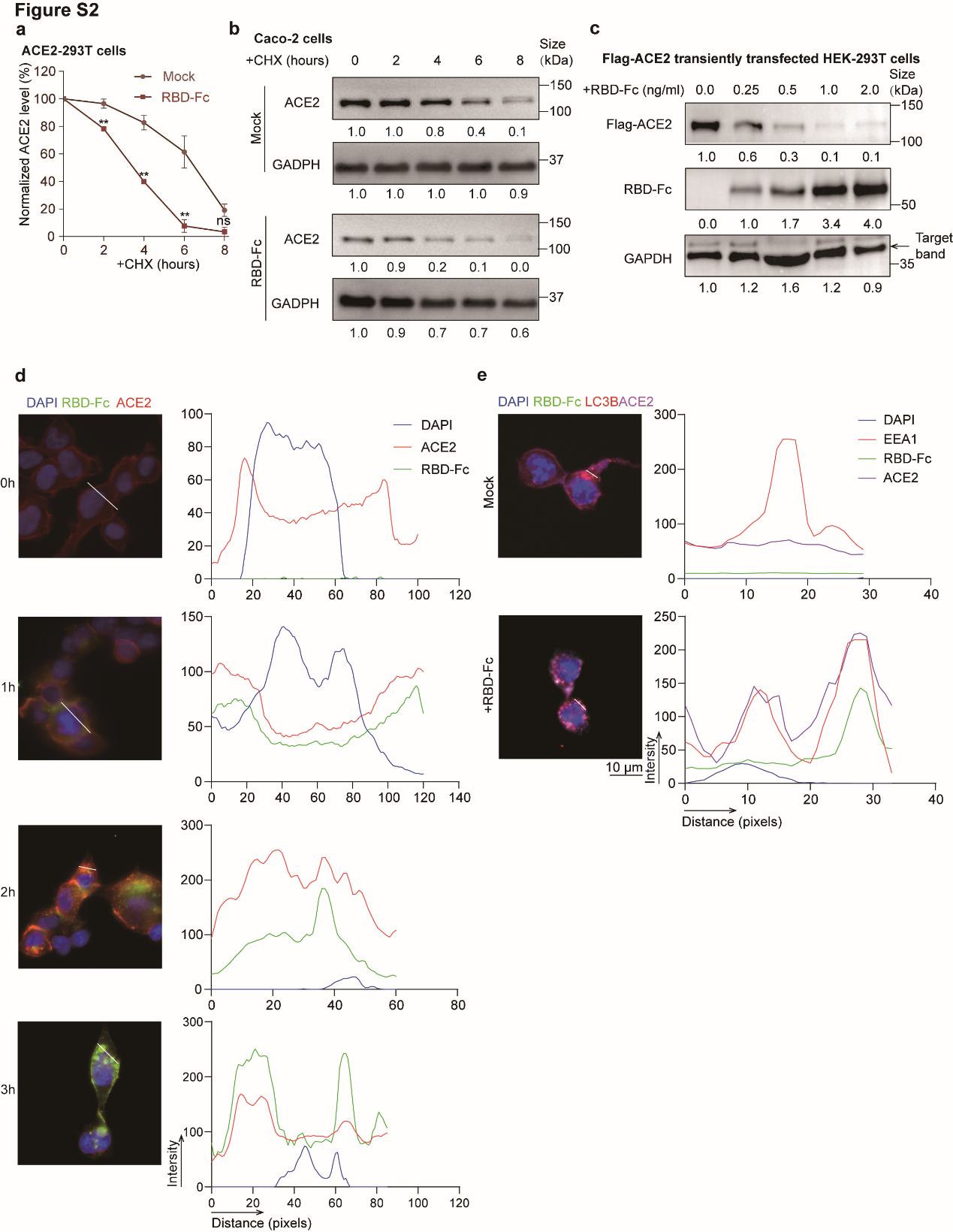


Figure. S2. Viral spike protein colocalized with ACE2 and mediated ACE2 endocytosis and degradation.

1. Statistic diagrams showing ACE2 degradation in **Figure 2a**. Band intensities of ACE2 relative to GAPDH were calculated and normalized to the ratio at 0-hour Protein levels (first lane). *P*-values were calculated by comparing the normalized ratio at the same timepoint with/without RBD-Fc treatment by paired T test. Error bars indicate SEM. ** *P* < 0.01. All unlabeled comparisons are not significant.
2. Immunoblots of extracts of Caco-2 cells treated with CHX (100 μg/ml) with or without RBD-Fc for 8 hours at 2-hour interval.
3. Immunoblots of extracts of ACE2 transiently transfected 293T cells treated with RBD-Fc for 6 hours at indicated concentration.
4. Immunofluorescence microscopy images showing the localization of ACE2 (red) in ACE2-293T cells treated with RBD-Fc (green) at indicated time points. Cell nuclei were stained with DAPI (Blue). Pixel intensity of the channels were plotted as indicated along the white line. Scale bars, 10 μm.
5. Immunofluorescence microscopy images showing the localization of ACE2 (magenta) and EEA1 (red) in ACE2-293T cells treated with RBD-Fc (green) at 1-hour post-treatment. Cell nuclei were stained with DAPI (Blue). Pixel intensity of the channels were plotted as indicated along the white line. Scale bars, 10 μm. Unless otherwise specified, *n*=3 biologically independent experiments were performed (**a-e**).


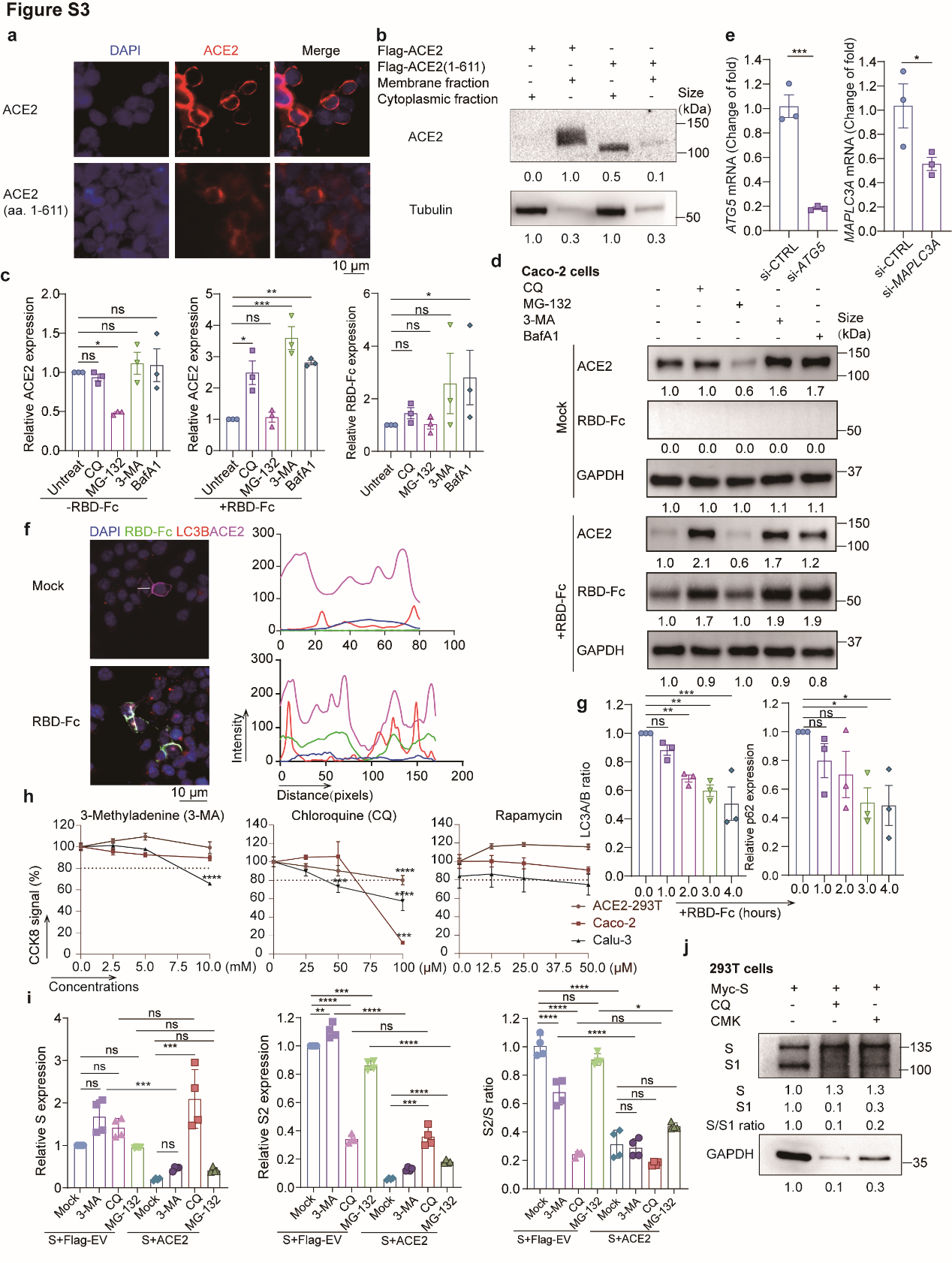


Figure. S3. Viral spike protein was degraded by autophagy.

1. Immunofluorescent microscopy images showing localization of Flag-ACE2 (Red) and Flag-ACE2(1-611) in transfected HEK-293T cells. Cell nuclei were stained with DAPI (Blue). Scale bars, 10 μm.
2. Immunoblot analysis of membrane and cytoplasmic fractions of HEK-293T cells transfected with Flag-ACE2 and Flag-ACE2(1-611).
3. Statistic diagrams showing relative protein levels of ACE2 and RBD-Fc in **Figure 3b**. Error bars indicate SEM. *P*-values were calculated by Dunnett’s multiple comparison test compared with untreated. * *P* < 0.05, ** *P* < 0.01; *** *P* < 0.001, ns: not significant.
4. Immunoblot images showing extracts of Caco-2 cells treated with mock or RBD-Fc in the presence of autophagy inhibitors (3-MA (5 mM), CQ (25 μM) or BafA1 (0.2 μM)) or a protease inhibitor (MG132 (10 μM)).
5. Validation of siRNA efficiency of *ATG5* and *MAP1LC3A* at 48-hour post-transfection. Error bars indicate SEM (n=3). *P*-values were calculated by paired T test. * *P* < 0.05, ** *P* < 0.01, *** *P* < 0.001, **** *P* < 0.0001.
6. Immunofluorescence microscopy images of the co-localization of ACE2 (magenta), LC3B (red) and RBD-Fc (green) in Flag-ACE2 transfected HEK293T cells with the treatment of RBD-Fc (green) at 3-hour post-treatment. Cell nuclei were stained with DAPI (Blue). Pixel intensity of the channels were plotted as indicated along the white line. Scale bars, 10 μm.
7. Statistic diagrams showing LC3A/B ratio and relative protein levels of p62 in **Figure 3e**. Error bars indicate SEM. *P*-values were calculated by Dunnett’s multiple comparison test with 0h. * *P* < 0.05, ** *P* < 0.01; *** *P* < 0.001, ns: not significant.
8. Concentration dependent chemical cytotoxicity of the autophagy inhibitors/ promoter (3-MA, CQ and rapamycin) measured by Cell Counting Kit-8 (CCK-8). Cells were treated with the chemicals at indicated concentration for 48 hours. CCK8 signals were normalized to the signal at 0 μM. Error bars indicate SEM (n = 4). * *P* < 0.05, ** *P* < 0.01, *** *P* < 0.001, **** *P* < 0.0001.
9. Statistic diagrams showing relative protein levels of S and S2 as well as S/S2 ratio in **Figure 3g**. Protein levels were normalized to Myc-S+Flag-EV with mock treatment (first lane). Error bars indicate SEM. *P*-values were calculated by Tukey’s multiple comparison test. * *P* < 0.05, ** *P* < 0.01; *** *P* < 0.001, **** *P* < 0.0001, ns: not significant.
10. Immunoblot analysis showing extracts of HEK-293T cells transfected with Myc-S, with the treatment of chloroquine (CQ) (50 μM) or CMK (50 μM). Unless otherwise specified, *n*=3 biologically independent experiments were performed (**a-j**).


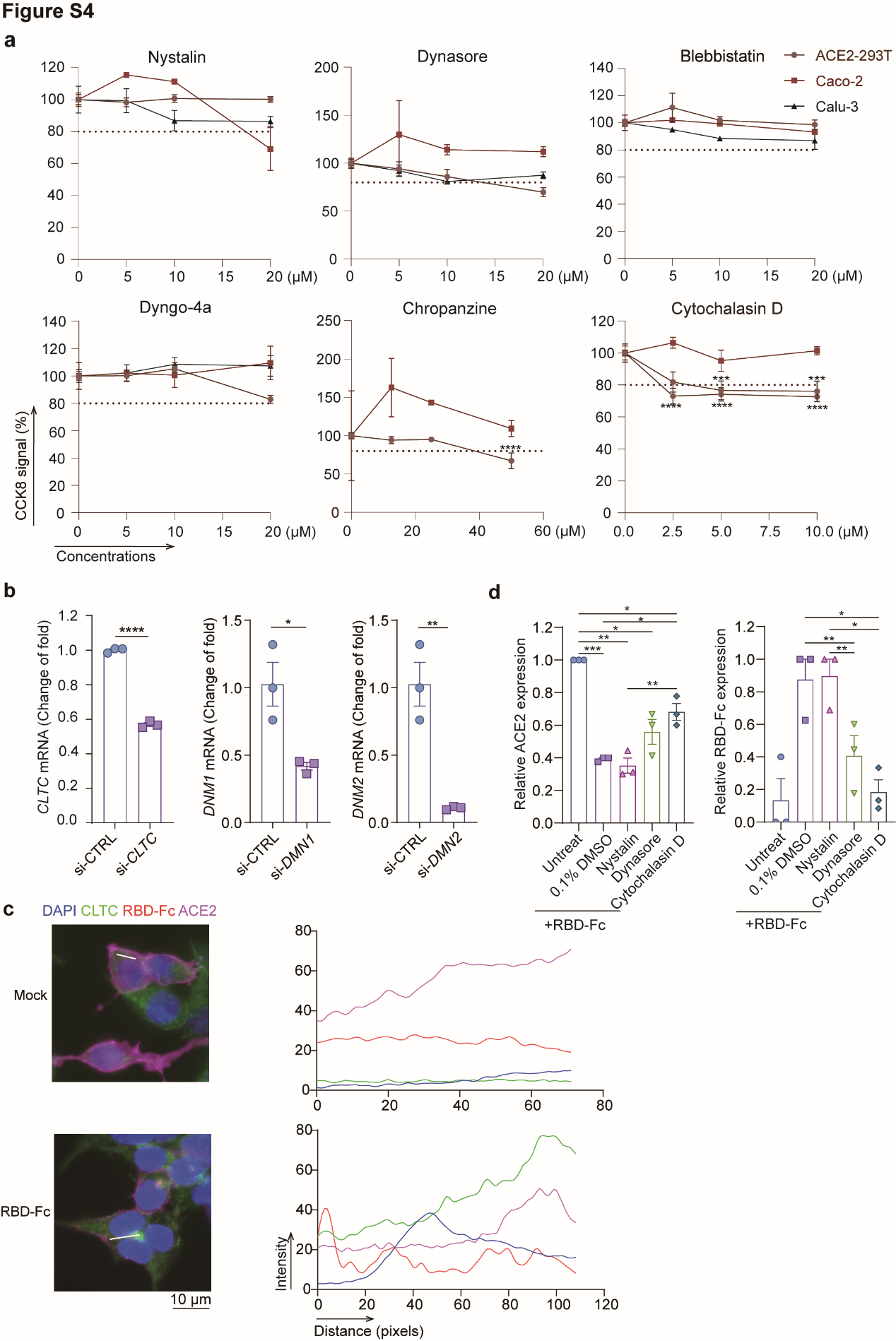


Figure. S4. Clathrin-mediated endocytosis played a critical role in ACE2 endocytosis.

1. Concentration dependent chemical cytotoxicity of the endocytosis inhibitors (nystalin, Dynasore, blebbisatin, dyngo-4a, chropanzine, and cytochalasin D) measured by Cell Counting Kit-8 (CCK-8). Cells were treated with the chemicals at indicated concentration for 48 hours. CCK8 signals were normalized to the signal at 0 μM. Error bars indicate SEM (n=4). * *P* < 0.05, ** *P* < 0.01, *** *P* < 0.001, **** *P* < 0.0001.
2. Validation of siRNA efficiency of *CLTC*, *DMN1* and *DMN2* at 48-hour post-transfection. Error bars indicate SEM (n=3). *P*-values were calculated by paired T test. * *P* < 0.05, ** *P* < 0.01, *** *P* < 0.001, **** *P* < 0.0001.
3. Immunofluorescence microscopy images of the co-localization of ACE2(magenta), CLTC (green) and S protein (red) in Flag-ACE2 transfected HEK293T cells at 3 hours post-infection of pseudovirus treatment. Cell nuclei were stained with DAPI (Blue). Pixel intensity of the channels were plotted as indicated along the white line. Scale bars, 10 μm.
4. Statistic diagrams showing relative protein levels of ACE2 and RBD-Fc. Error bars indicate SEM. *P*-values were calculated by Tukey’s multiple comparison test. * *P* < 0.05, ** *P* < 0.01; *** *P* < 0.001. All unlabeled comparisons are not significant. Unless otherwise specified, *n*=3 biologically independent experiments were performed (**a-d**).

**
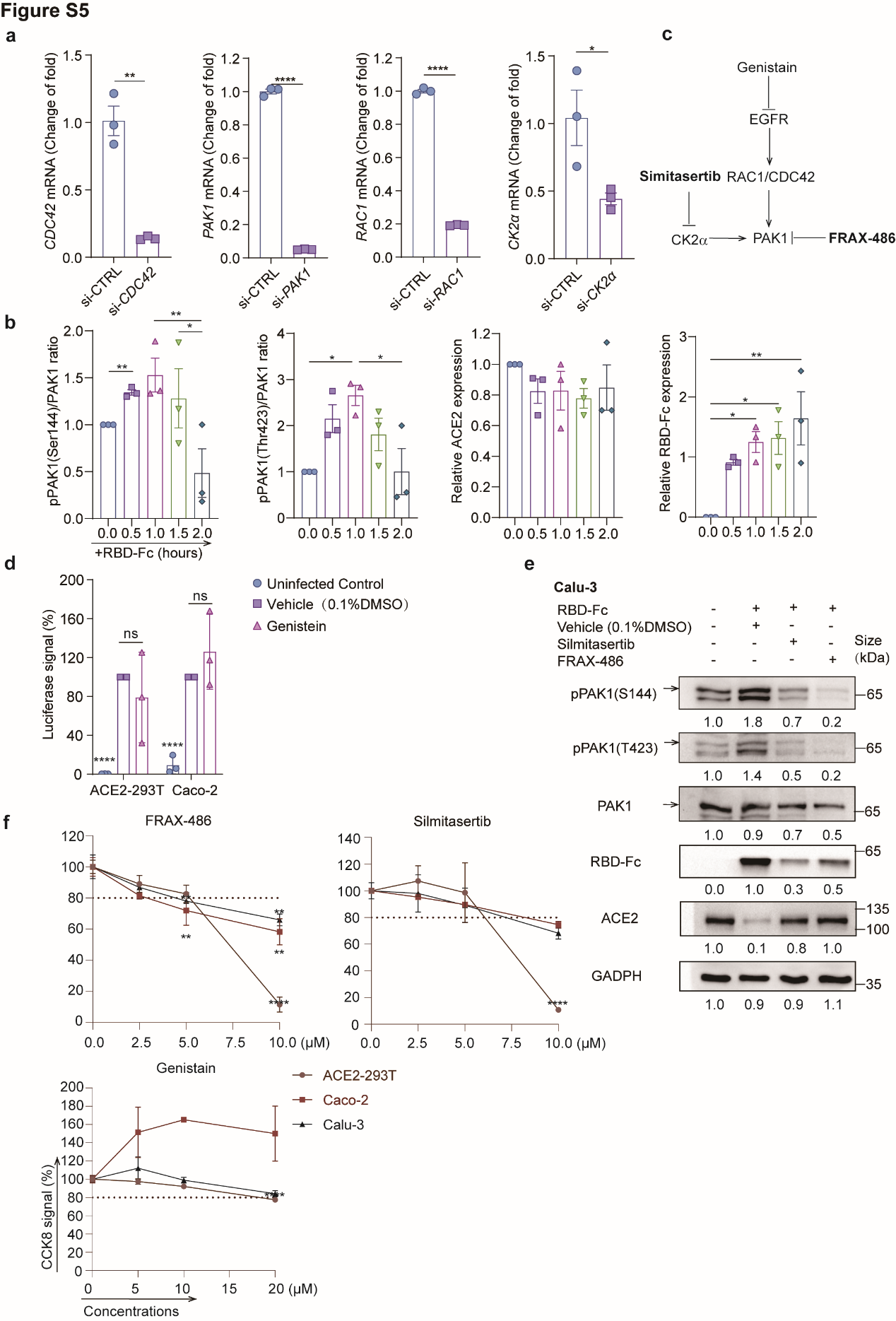
**

**Figure. S5. The PAK1 activation signaling pathway.**

1. Validation of siRNA efficiency of the indicated genes 48-hour post-transfection. Error bars indicate SEM (n=3). *P*-values were calculated by paired T test. * *P* < 0.05, ** *P* < 0.01, *** *P* < 0.001, **** *P* < 0.0001.
2. Statistic diagrams showing pPAK1(Ser144)/PAK1 ratio, pPAK1(Thr423)/PAK1 ratio, and relative protein levels of ACE2 and RBD-Fc. Error bars indicate SEM. *P*-values were calculated by Tukey’s multiple comparison test. * *P* < 0.05, ** *P* < 0.01. All unlabeled comparisons are not significant.
3. The signaling pathway and inhibitors of PAK1 activation.
4. Entry of SARS-CoV-2 pseudovirus into ACE2-293T and Caco-2 cells with the EGFR inhibitor Genistain (20 μM). Viral entry efficiency was taken as the percentage of luciferase signal with inhibitors/positive control. Error bars indicate SEM (n=3). **** *P* < 0.0001, ns: not significant.
5. Immunoblot analysis of extracts of RBD-Fc treated Calu-3 cells incubated with or vehicle (0.1% DMSO), FRAX-486 (2.5 μM) or silmitasertib (2.5 μM).
6. Concentration dependent chemical cytotoxicity of the chemicals measured by Cell Counting Kit-8 (CCK-8). Cells were treated with the chemicals at indicated concentration for 48 hours. CCK8 signals were normalized to the signal at 0 μM. Error bars indicate SEM (n=4). Error bars indicate SEM (n=4). * *P* < 0.05, ** *P* < 0.01, *** *P* < 0.001, **** *P* < 0.0001. Unless otherwise specified, *n*=3 biologically independent experiments were performed (**a-f**).


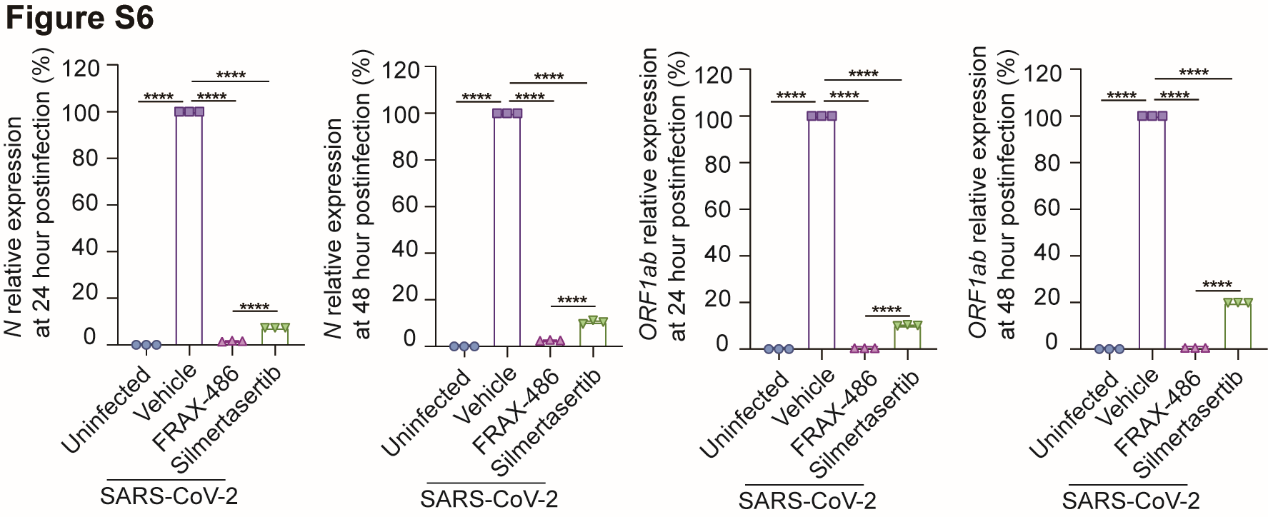


Figure. S6. Relative viral RNA (N and *ORF1ab*) expression level with FRAX-486 (2.5 μM), silmitasertib (2.5 μM) or vehicle (0.1% DMSO) at 24- and 48-hours SARS-CoV-2 post-infection. Error bars indicate SEM (n=3). *P*-values were calculated by Tukey’s multiple comparison test. **** *P* < 0.0001.

Table S1. Chemical list.

| Chemical name | Resources | Working concentration |
| --- | --- | --- |
| Camostat | Selleck, Cat#S2874 | 50 μM |
| Nystatin | Selleck, Cat#S1934 | 10 μM |
| Dynasore | Selleck, Cat#S1934 | 20 μM |
| Cytochalasin D | Target Mol, Cat# 22144-77-0 | 10 μM |
| Cycloheximide (CHX) | Sigma-Aldrich, Cat#C1988 | 100 μg/ml (1) |
| 3-Methyladenine (3-MA) | Sigma-Aldrich, Cat#M9281 | 5 mM for 48 hour;  10 mM for 6 hour (1) |
| Chloroquine (CQ) | Sigma-Aldrich, Cat#PHR1258 | 25 μM for 48 hour;  50 μM for 6 hour (1) |
| Bafilomycin A1(BafA1) | MCE, Cat# HY-100558 | 0.2 μM for 6 hour (1) |
| MG-132 | Sigma-Aldrich, Cat#C2211 | 10 μM (1) |
| NH_4_Cl | Sigma, Cat#A9434 | 2 mM |
| Silmitasertib | Target Mol, Cat#T2259 | 2.5 μM |
| FRAX486 | Target Mol, Cat#T6840 | 2.5 μM |
| Dyngo-4a | Selleck, Cat#S7163 | 20 μM |
| Chlorpromazine | Selleck, Cat#S7163 | 25 μM |
| Rapamycin | Selleck, Cat#S1039 | 50 μM |
| Blebbistatin | Selleck, Cat#S7099 | 20 μM |
| Genistein | Target Mol, Cat# T1737 | 20 μM |
| decanoyl-RVKR-chloromethylketone (CMK) | Tocris, Cat# 3501 | 50 μM |

Table S2. siRNA list.

| Gene | SiRNA sequences | References |
| --- | --- | --- |
| LC3 | 5’- GUAGAAGAUGUCCGACUUATTdTdT | - |
| ATG5 | 5’- UUCAUGGAAUUGAGCCAAUTTdTdT | - |
| CLHC | 5’-UAAUCCAAUUCGAAGACCAAUdTdT | (2) |
| PAK1 | 5’-GCGAUCCUAAGAAGAAAUAUACAdTdT | (3) |
| RAC1 | 5’-GGAACUAAACUUGAUCUUAGGGAdTdT | (3) |
| CDC42 | 5’-CCACAAACAGAUGUAUUUCUAGUdTdT | (3) |
| CK2α | 5’-GAUGACUACCAGCUGGUUCdTdT | (4) |
| DNM1 | 5’-GGCUUACAUGAACACCAACCACGAAdTdT | (5) |
| DNM2-1 | 5’-CCGAAUCAAUCGCAUCUUCUUdTdT | (5) |
| DNM2-2 | 5’-GACAUGAUCCUGCAGUUCAUUdTdT | (5) |

Table S3 primer list for RT-PCR.

| Gene | Primer |
| --- | --- |
| *CK2*α | Forward: TCCCGAGCTGGGGTAATCAA  Reverse: GTTCCACCACGAAGGTTCTCC |
| *ACE2* | Forward: CGAAGCCGAAGACCTGTTCTA  Reverse: GGGCAAGTGTGGACTGTTCC |
| *PAK1* | Forward: CAGCCCCTCCGATGAGAAATA  Reverse: CAAAACCGACATGAATTGTGTGT |
| *CLTC* | Forward: ATTCTGCCAATTCGTTTTCAGGA  Reverse: GCTTTCAGTGCAATTACTTTGCT |
| *RAC1* | Forward: ATGTCCGTGCAAAGTGGTATC  Reverse: CTCGGATCGCTTCGTCAAACA |
| *CDC42* | Forward: CCATCGGAATATGTACCGACTG  Reverse: CTCAGCGGTCGTAATCTGTCA |
| *DNM1* | Forward: ATATGCCGAGTTCCTGCACTG  Reverse: AGTAGACGCGGAGGTTGATAG |
| *DNM2* | Forward: CCGCTGGTCAACAAACTGC  Reverse: CGGGTGACGATTCCTGAACC |
| *GADPH* | Forward: GGAGCGAGATCCCTCCAAAAT  Reverse: GGCTGTTGTCATACTTCTCATGG |
| SARS-COV-2 (*N*) | Forward: GGGGAACTTCTCCTGCTAGAAT  Reverse: CAGACATTTTGCTCTCAAGCTG |
| SARS-COV-2 (*ORF1ab*) | Forward: CCCTGTGGGTTTTACACTTAA  Reverse: ACGATTGTGCATCAGCTGA |
| *LC3* | Forward: AACATGAGCGAGTTGGTCAAG  Reverse: GCTCGTAGATGTCCGCGAT |
| *ATG5* | Forward: AAAGATGTGCTTCGAGATGTGT  Reverse: CACTTTGTCAGTTACCAACGTCA |

References

1. S. Jin *et al.*, USP19 modulates autophagy and antiviral immune responses by deubiquitinating Beclin-1. *EMBO J* **35**, 866-880 (2016).

2. A. Motley, N. A. Bright, M. N. Seaman, M. S. Robinson, Clathrin-mediated endocytosis in AP-2-depleted cells. *J Cell Biol* **162**, 909-918 (2003).

3. H. Costa Verdera, J. J. Gitz-Francois, R. M. Schiffelers, P. Vader, Cellular uptake of extracellular vesicles is mediated by clathrin-independent endocytosis and macropinocytosis. *J Control Release* **266**, 100-108 (2017).

4. K. Yamane, T. J. Kinsella, CK2 inhibits apoptosis and changes its cellular localization following ionizing radiation. *Cancer Res* **65**, 4362-4367 (2005).

5. C. R. Reis, P. H. Chen, N. Bendris, S. L. Schmid, TRAIL-death receptor endocytosis and apoptosis are selectively regulated by dynamin-1 activation. *Proc Natl Acad Sci U S A* **114**, 504-509 (2017).

Data S1. (separate file)

All original films of Western blots.
