# Supplementary material for: P21-activated kinase 1 (PAK1)-mediated cytoskeleton rearrangement promotes SARS-CoV-2 entry and ACE2 autophagic degradation
Source: Signal Transduct Target Ther. 2023 Oct 9;8:385. doi: 10.1038/s41392-023-01631-0 (PMC10560660; doi:10.1038/s41392-023-01631-0)
Supplement: Supplementary file 2 — Data S1. All original films of Western blots [file 41392_2023_1631_MOESM2_ESM.pdf]

Figure 1a

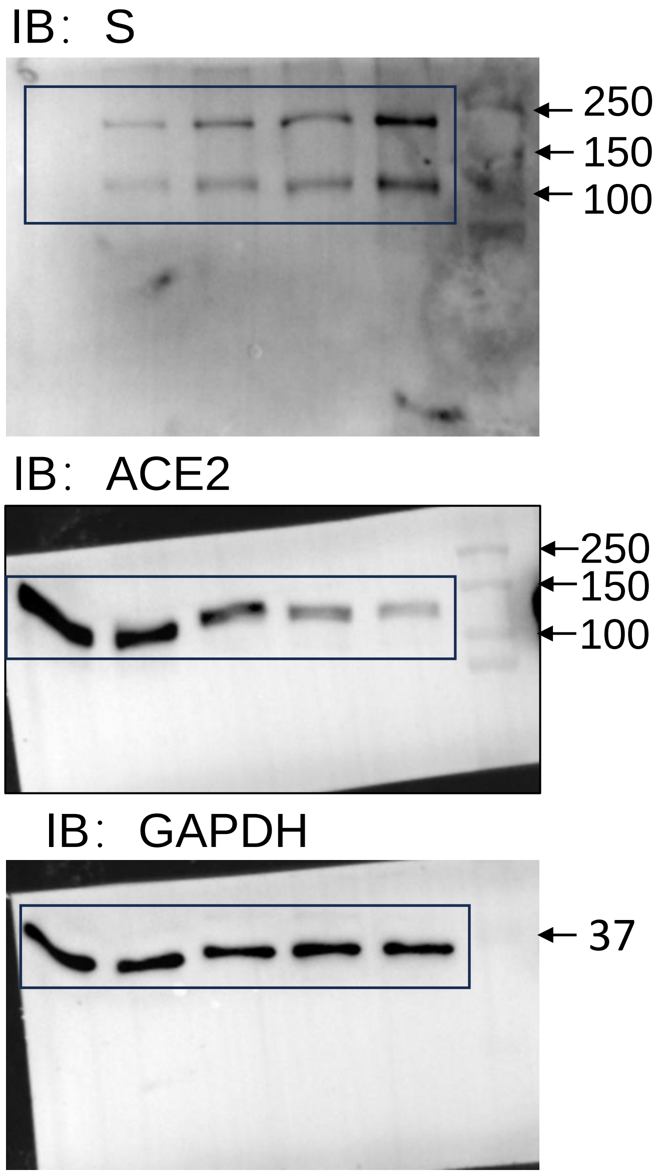

Figure 1b

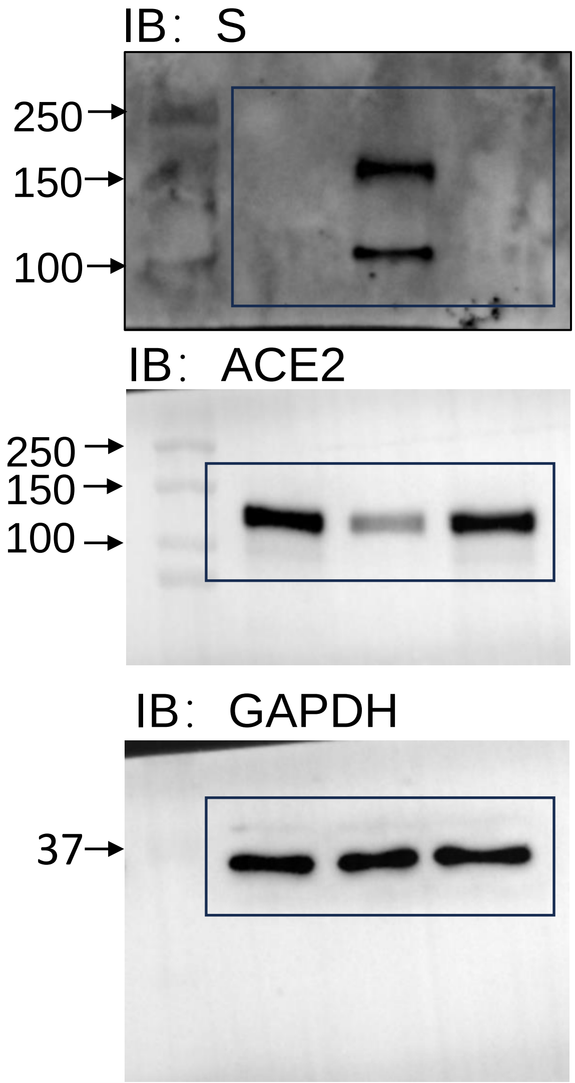

Figure 1c

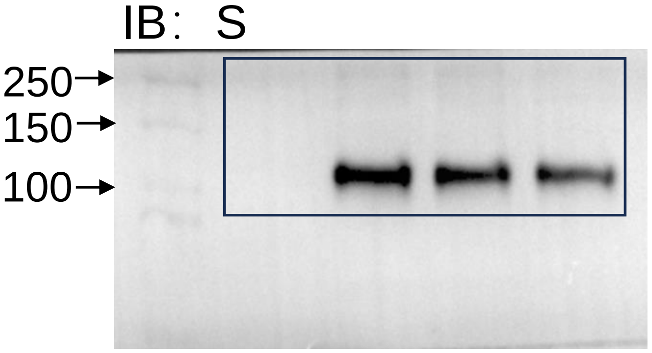

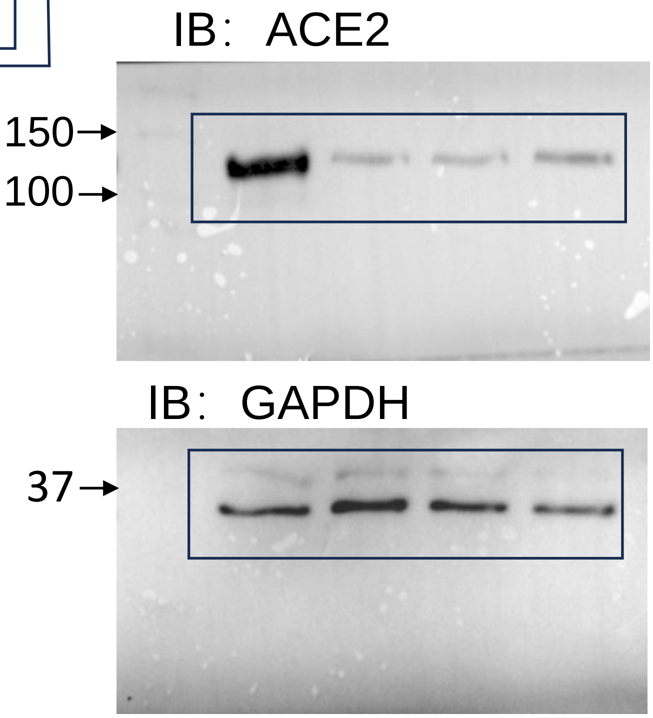

Figure 1e

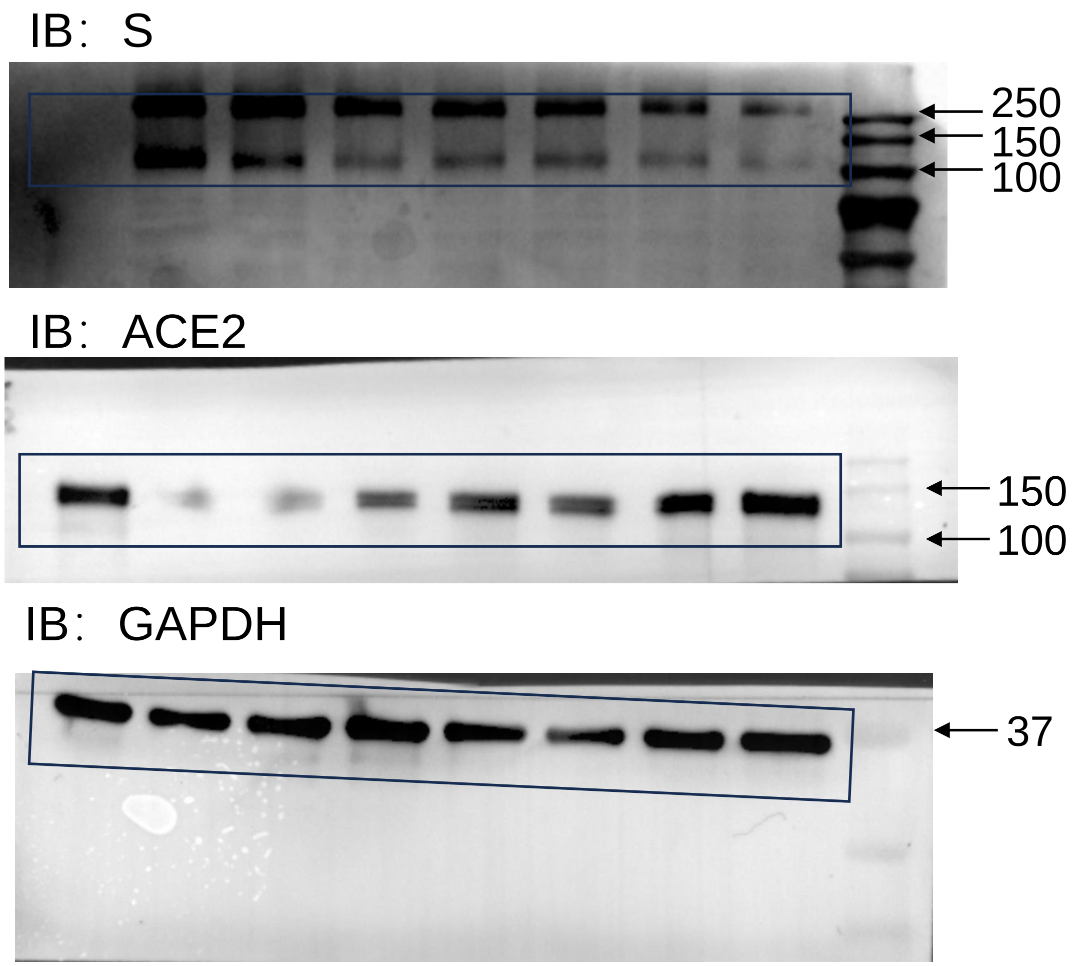

Figure 2a

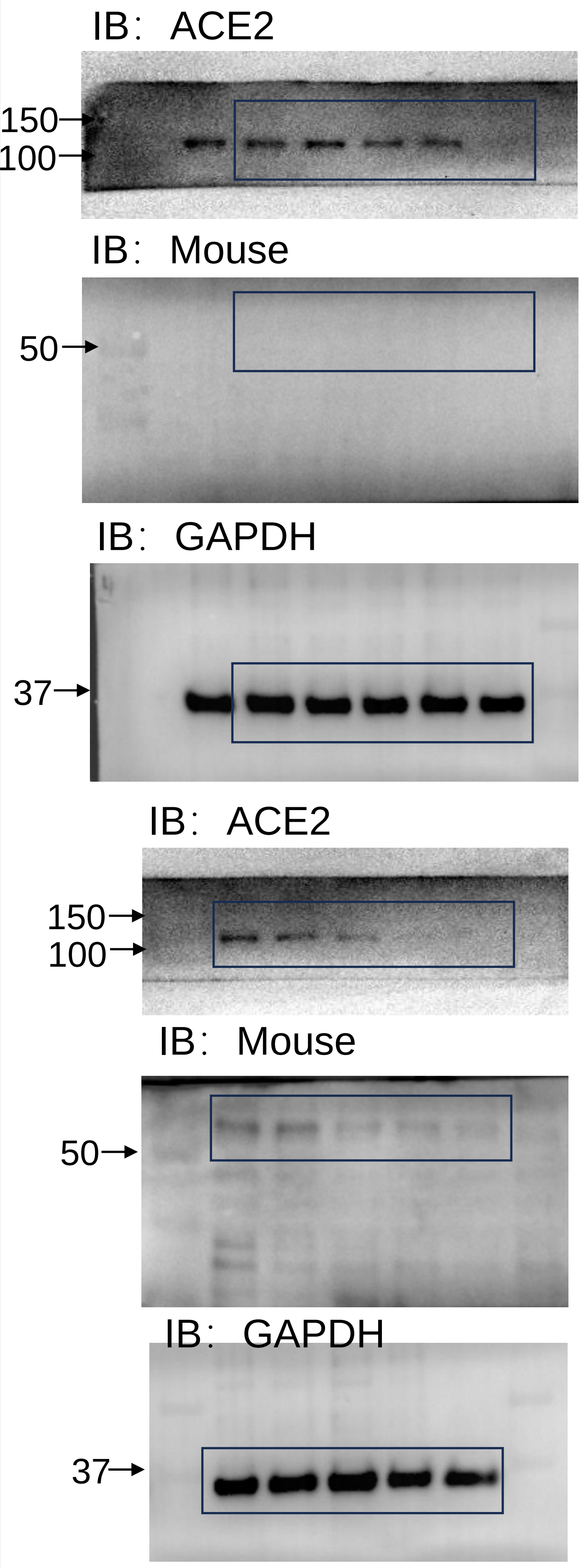

Figure 2b

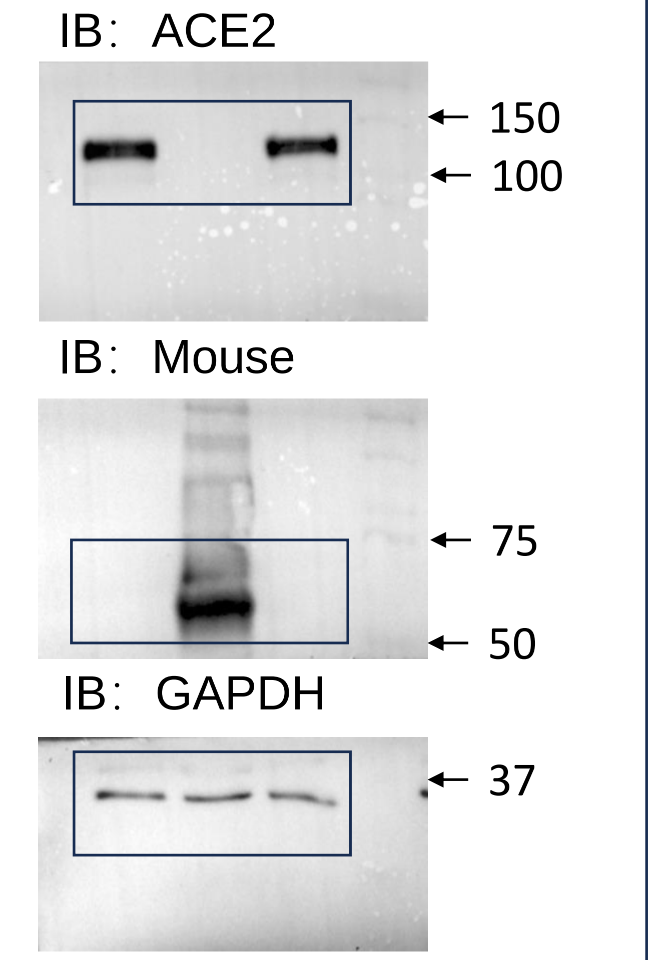

Figure 2c

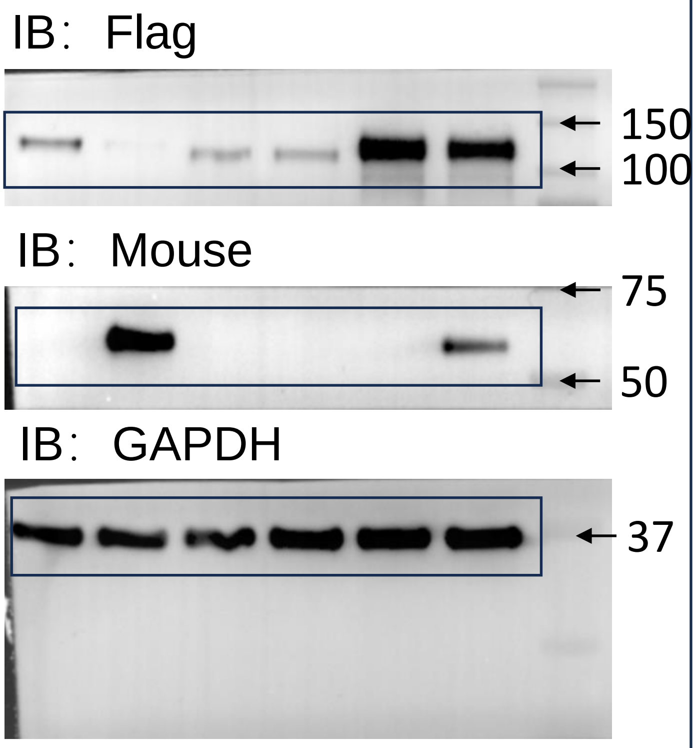

Figure 3a

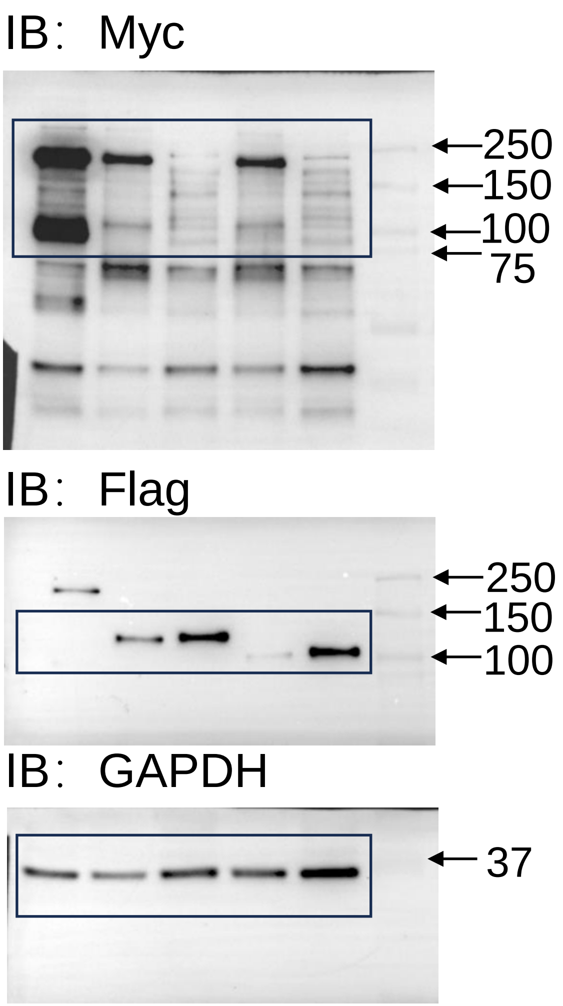

Figure 3c

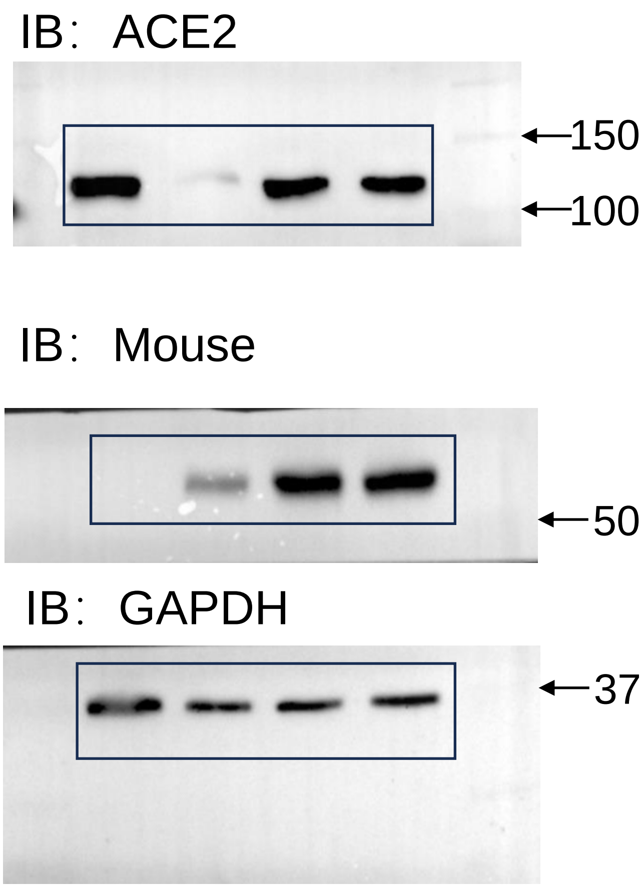

Figure 3b

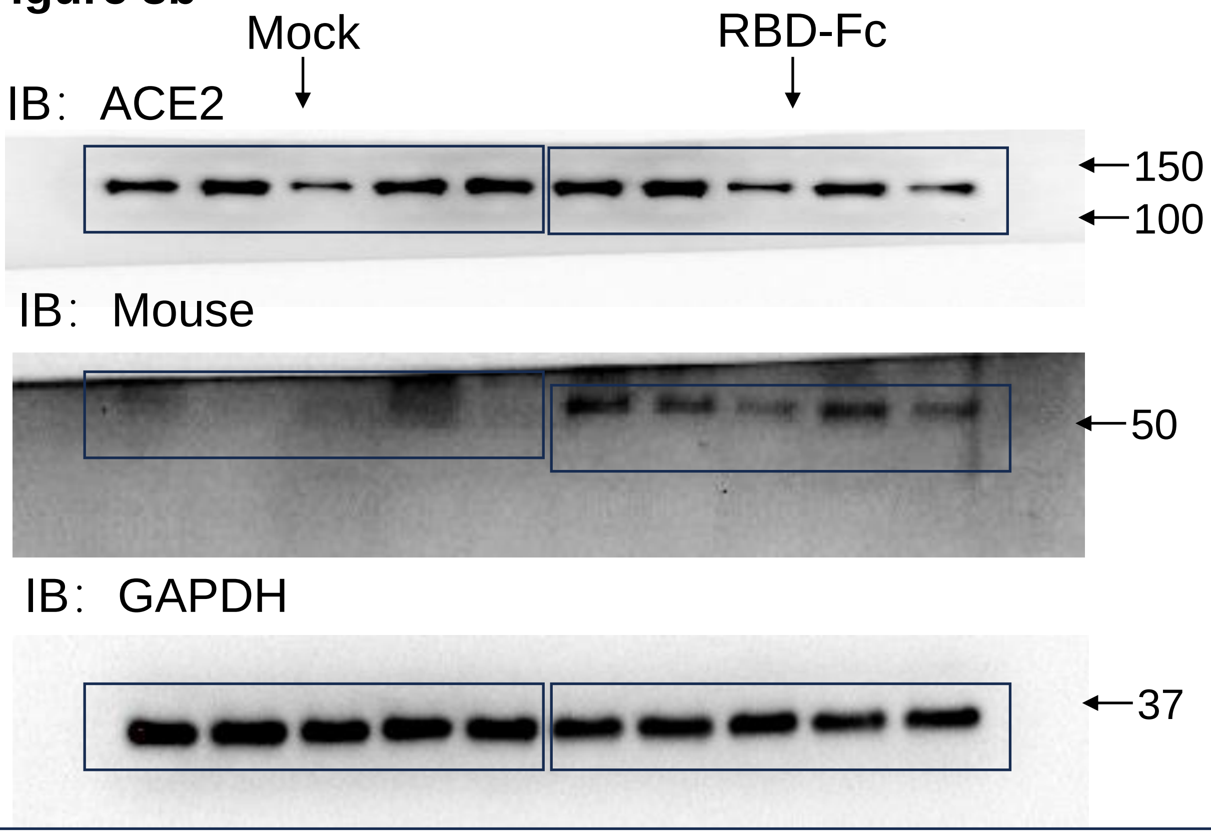

Figure 3e

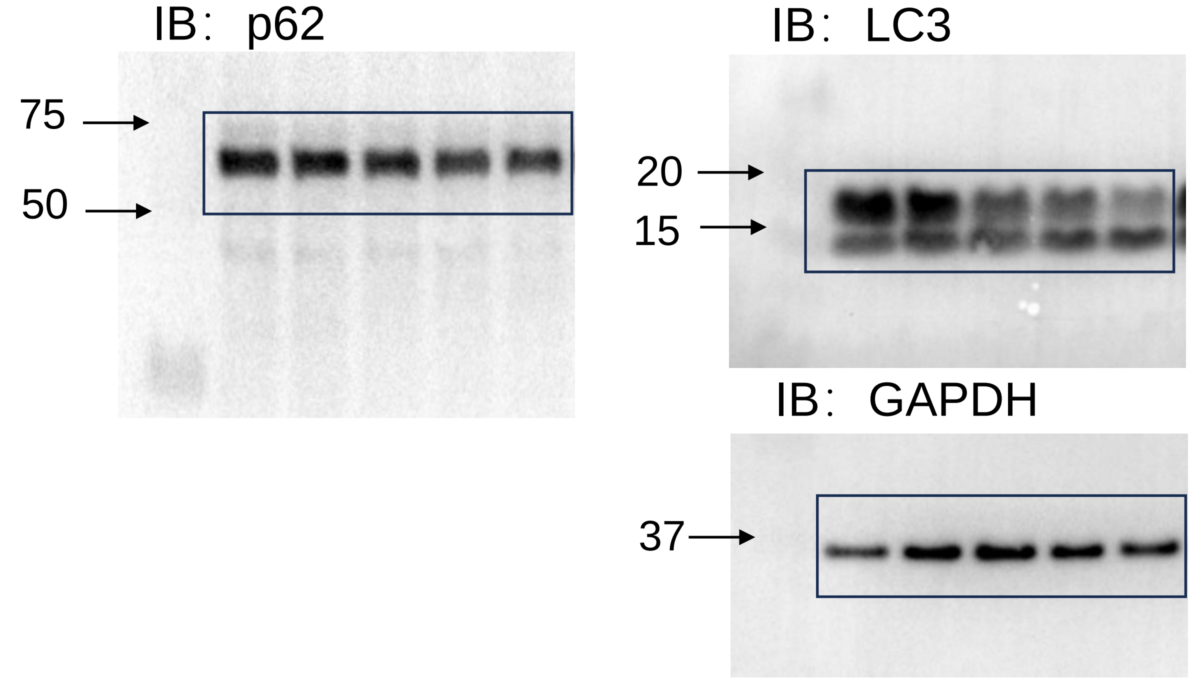

Figure 3g

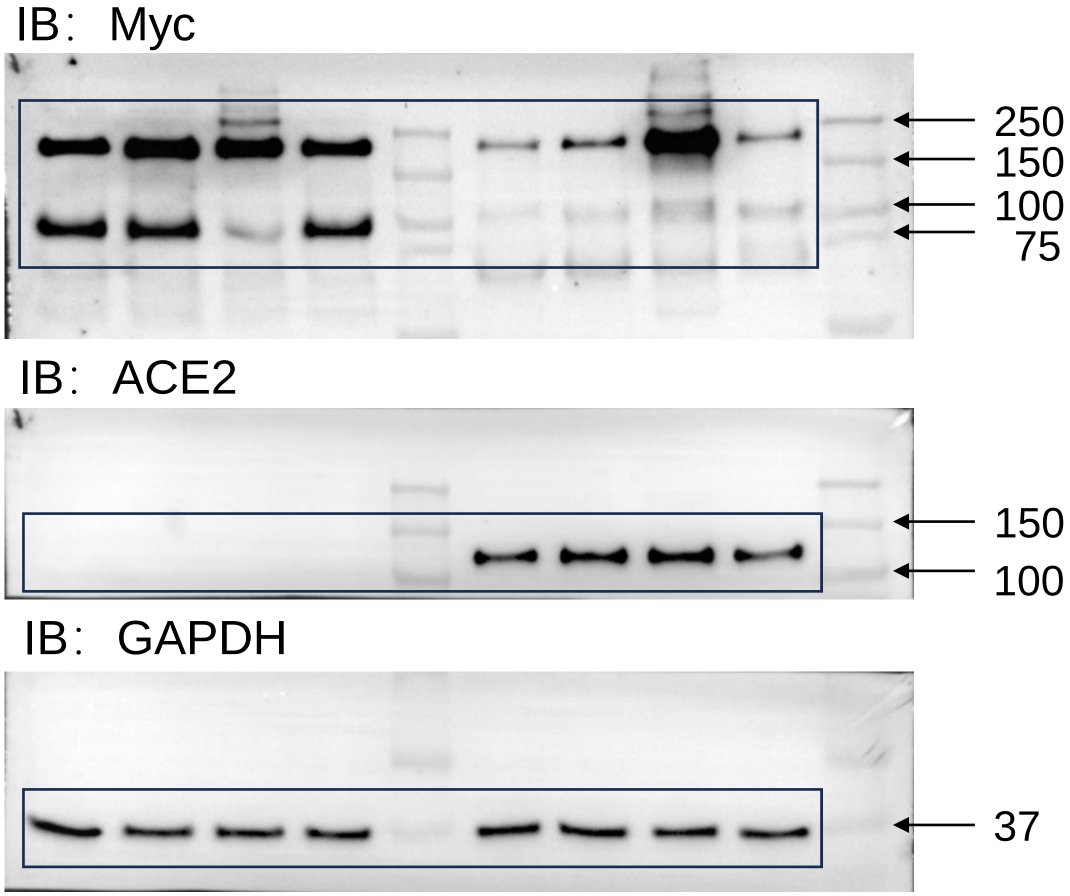

Figure 4f

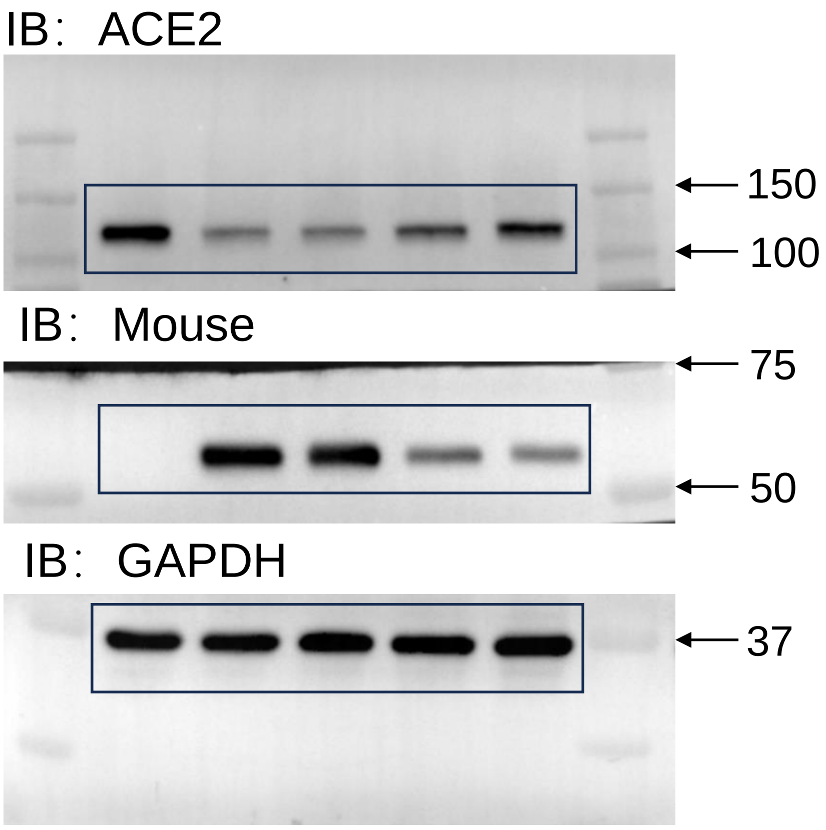

Figure 5b

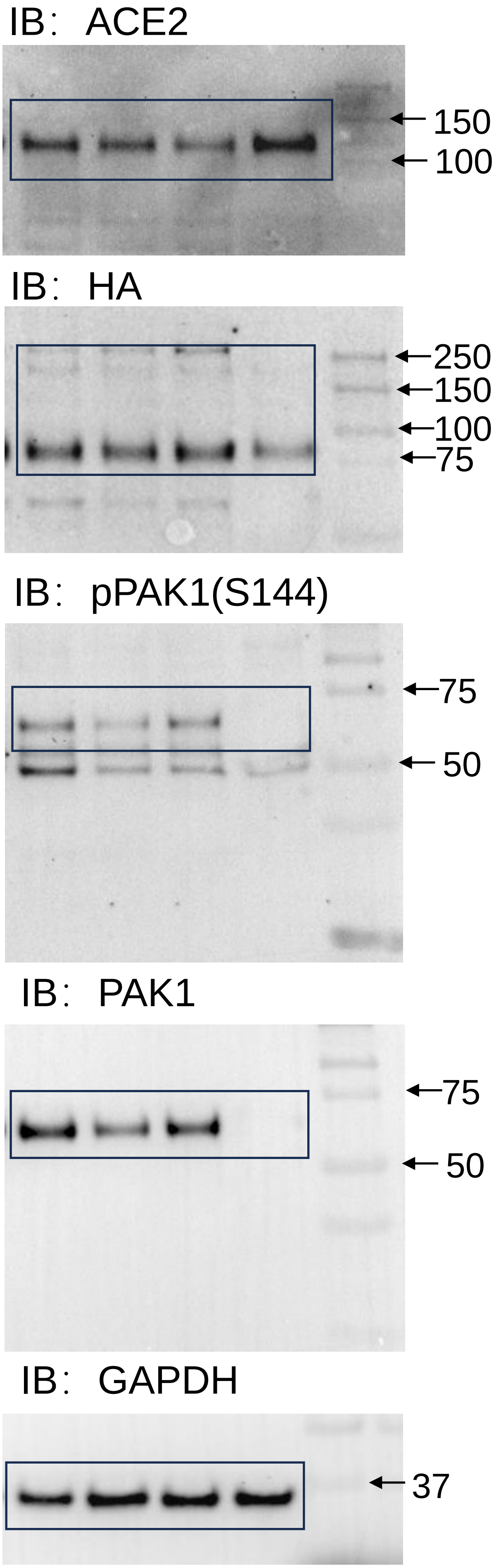

Figure 5c

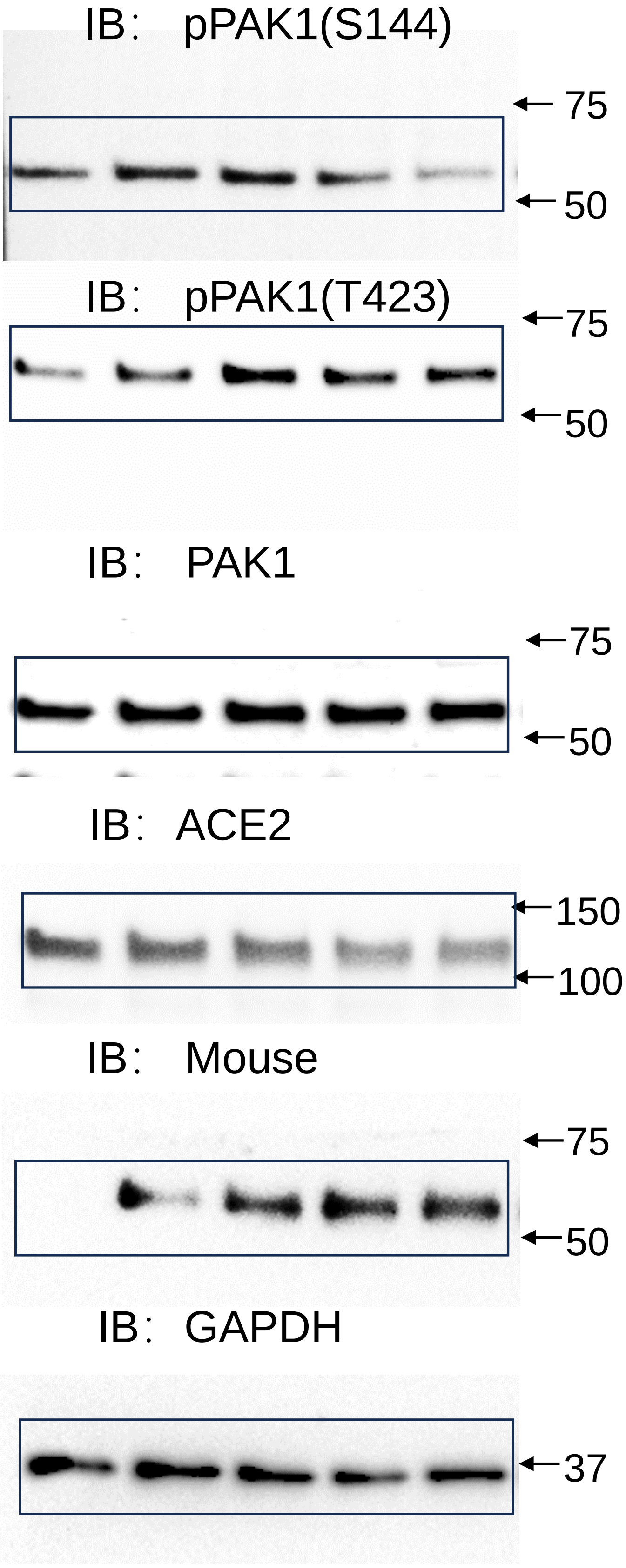

Figure 5d

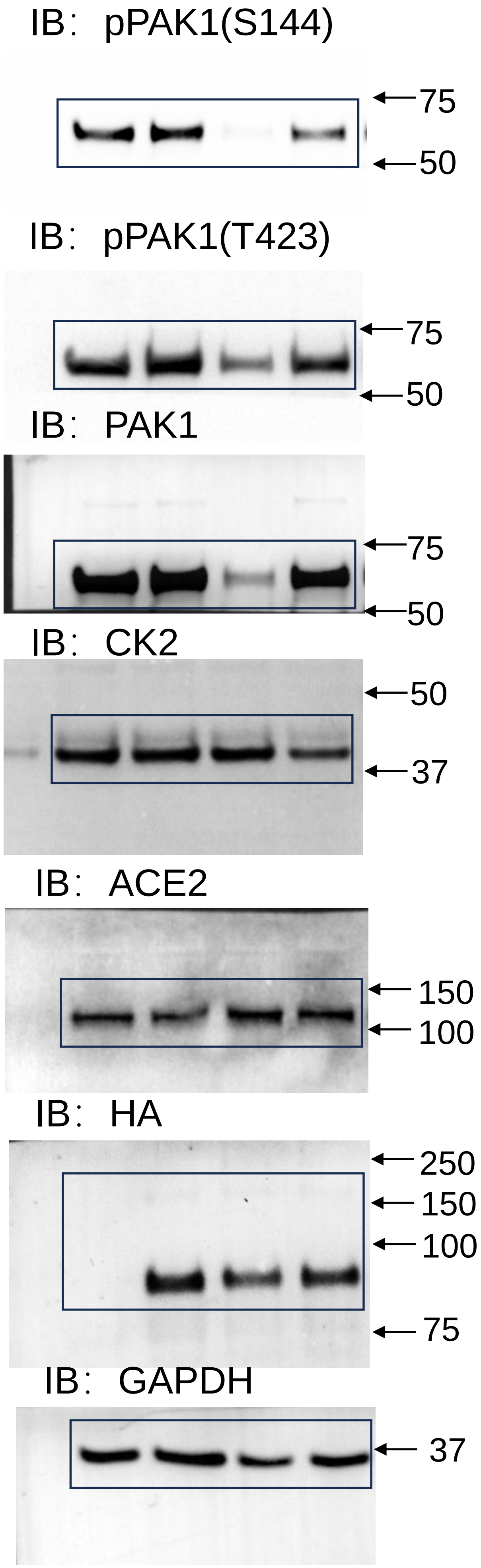

Figure 5g

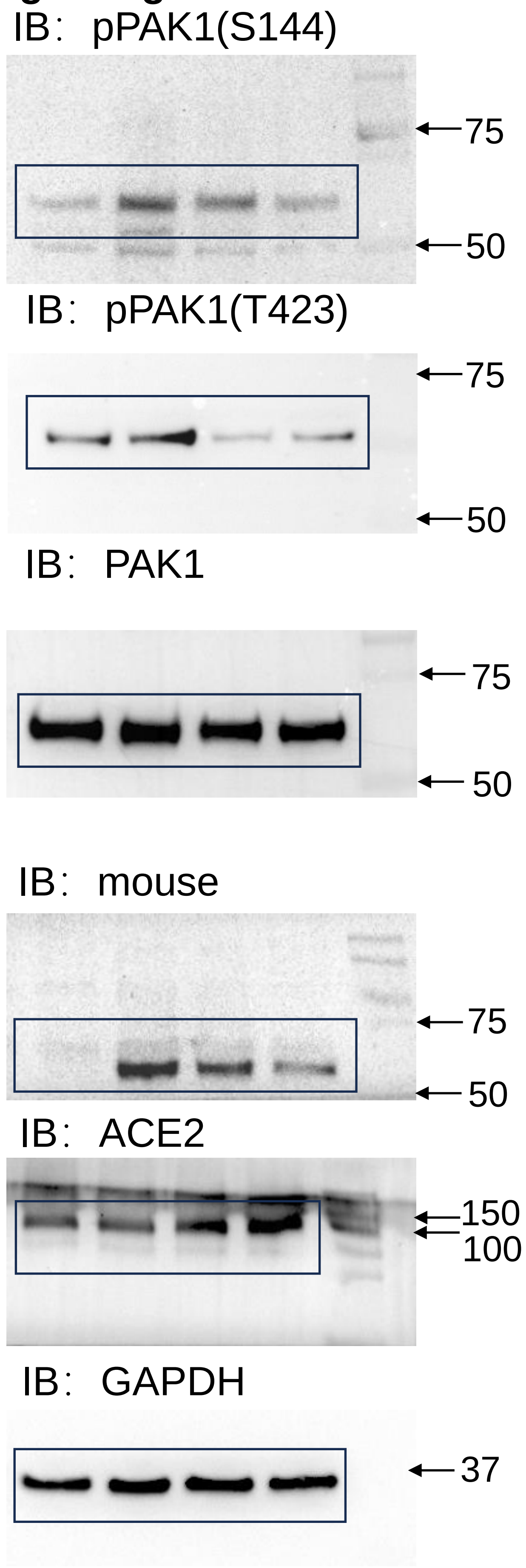

Figure 6b

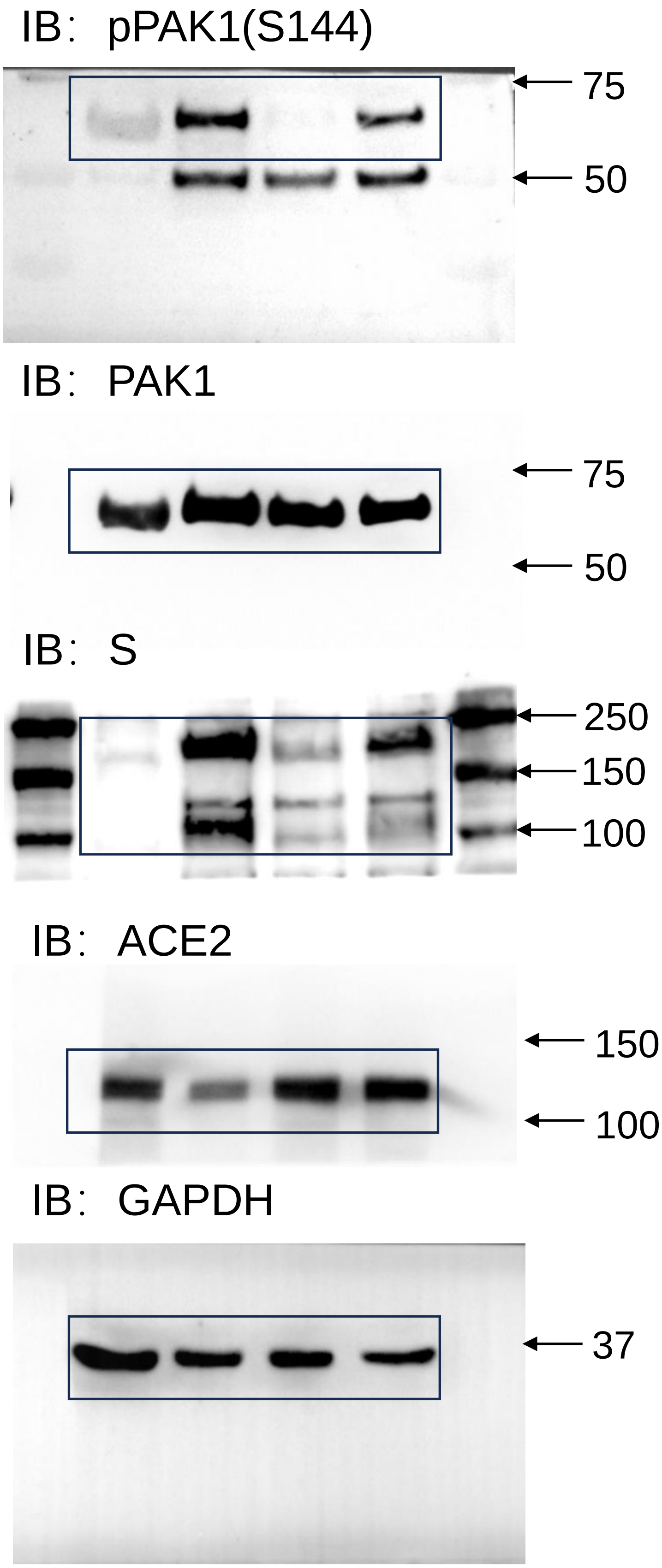
IB: pPAK1(S144)
75
50
IB: PAK1
75
50
IB: S
250
150
100
IB: ACE2
150
100
IB: GAPDH
37

sFigure 1b

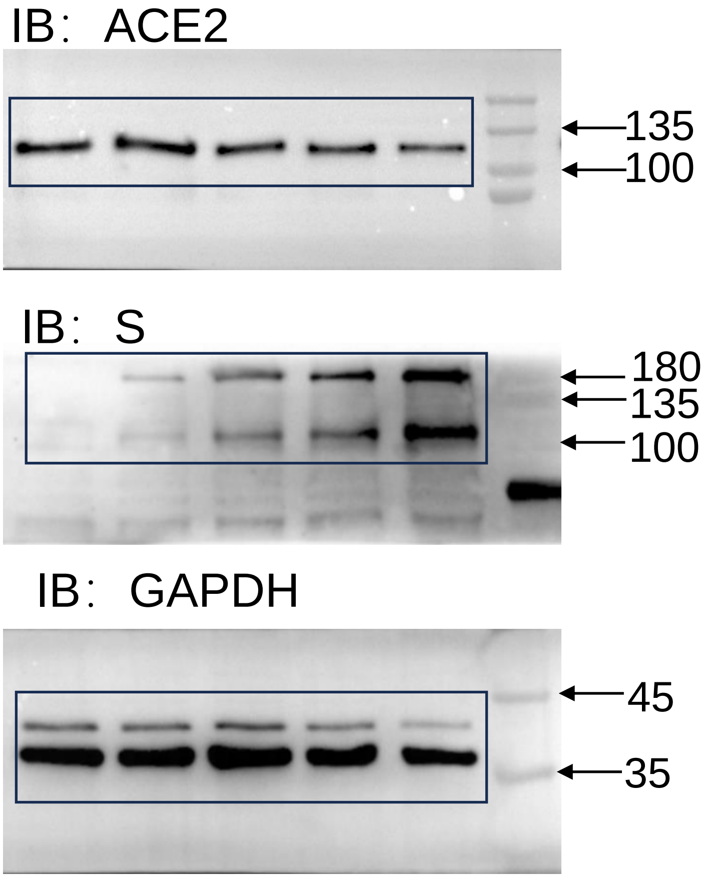

sFigure 1e

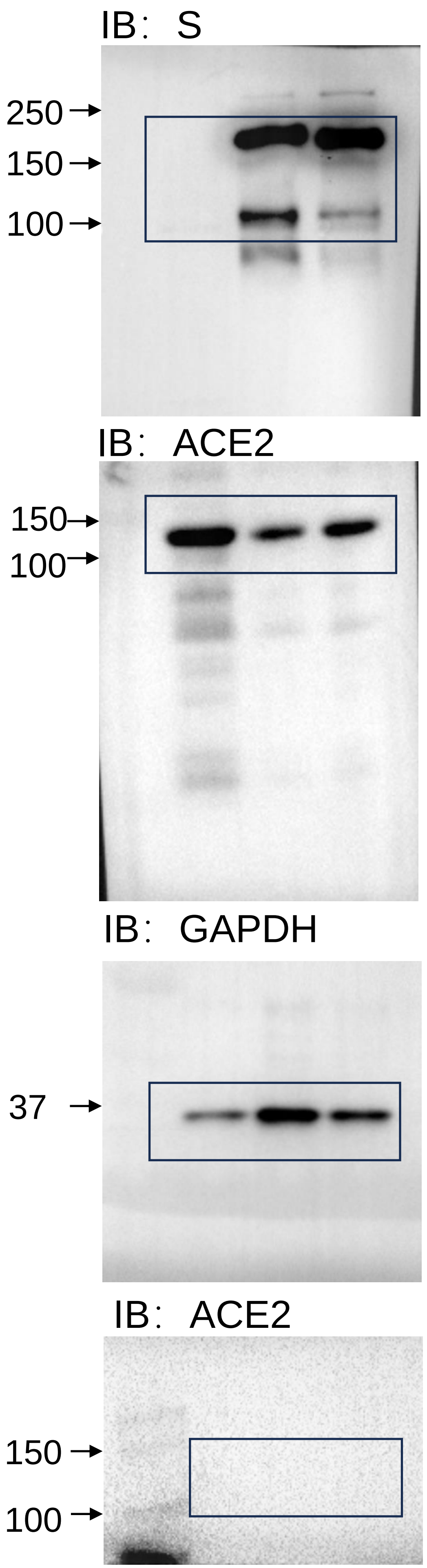

sFigure 2b

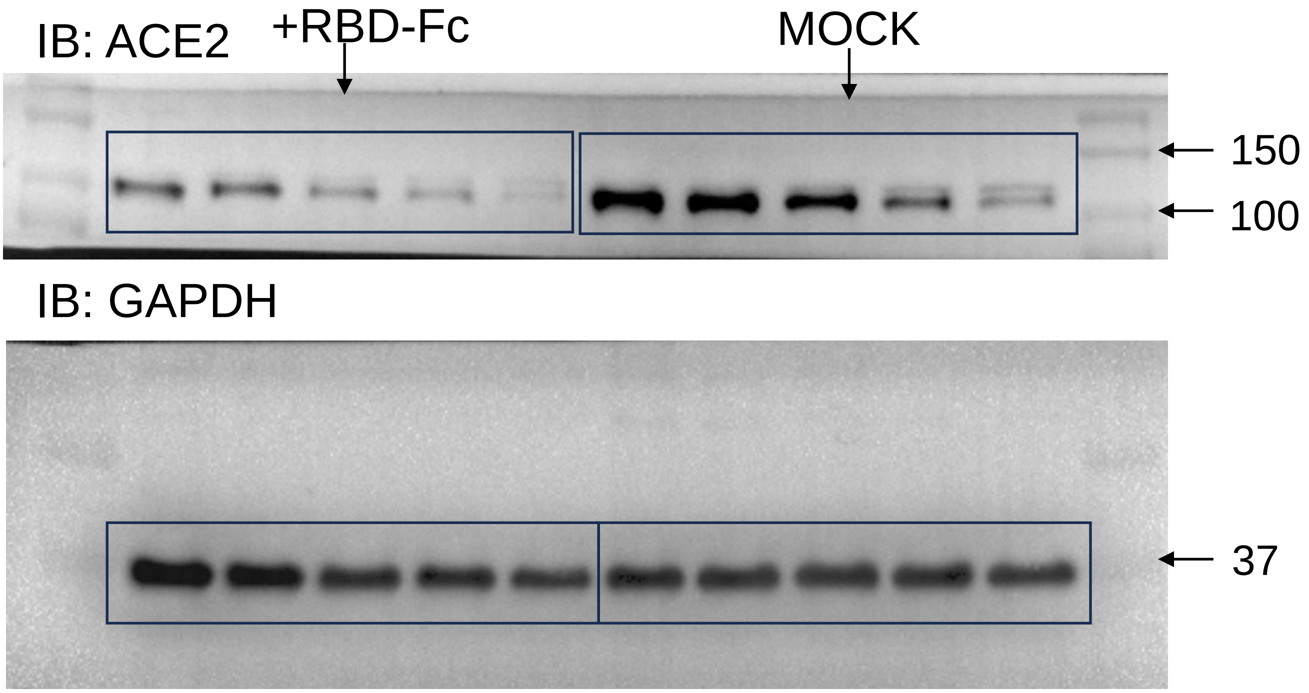

sFigure 2c

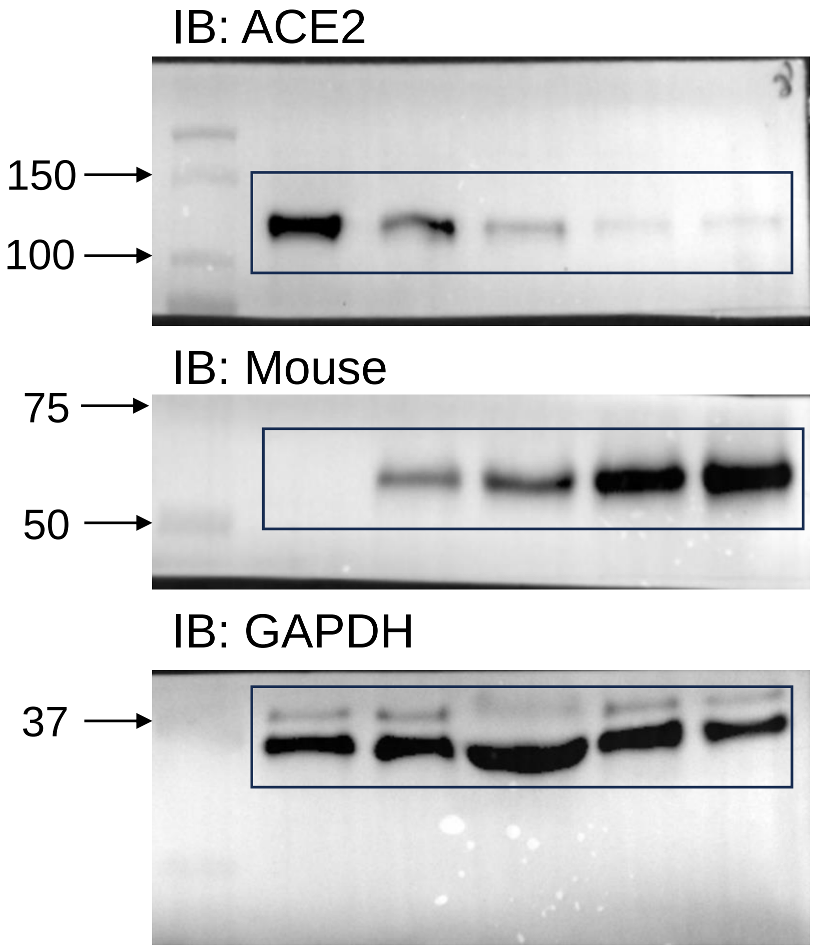

sFigure 3b

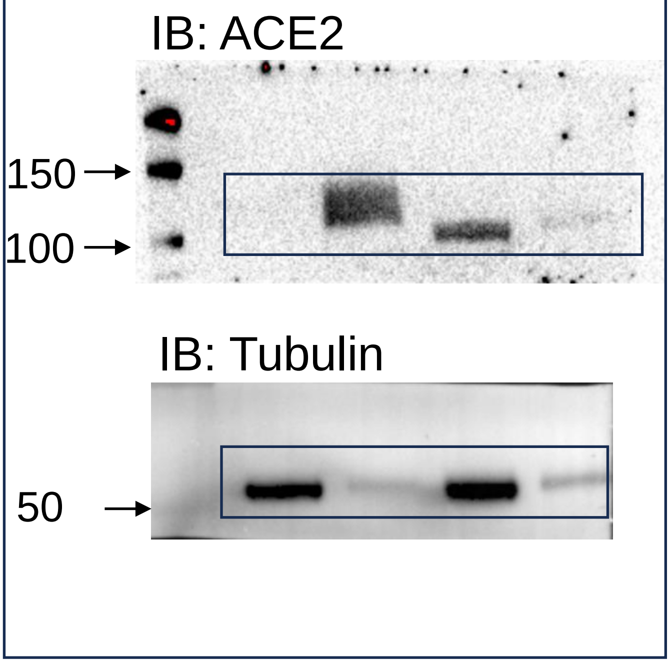

sFigure 3j

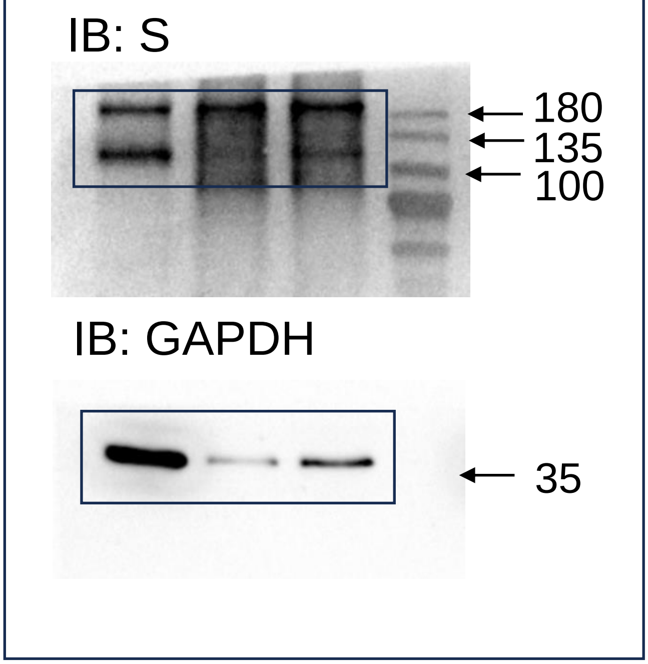

sFigure 3d

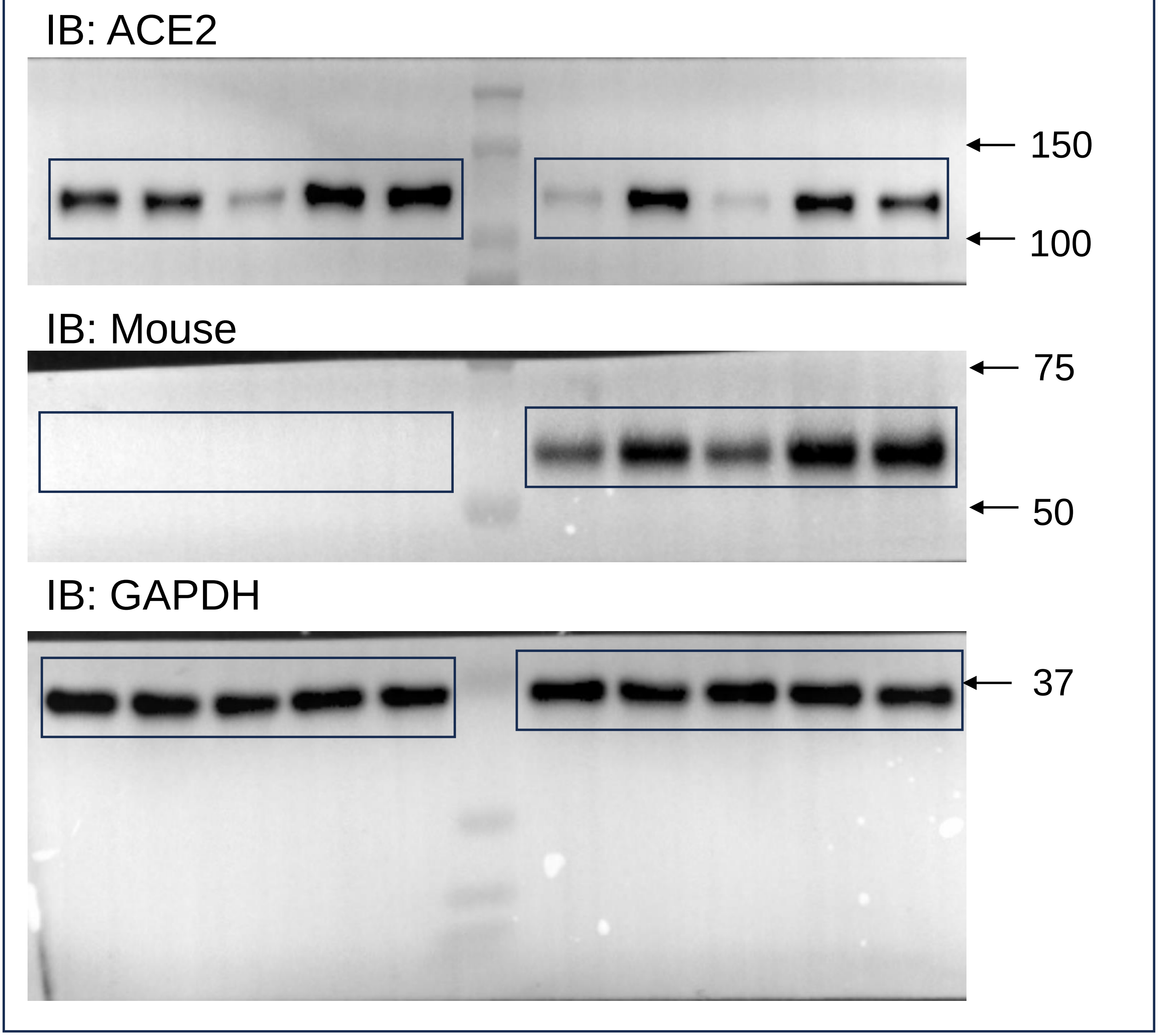

sFigure 5e

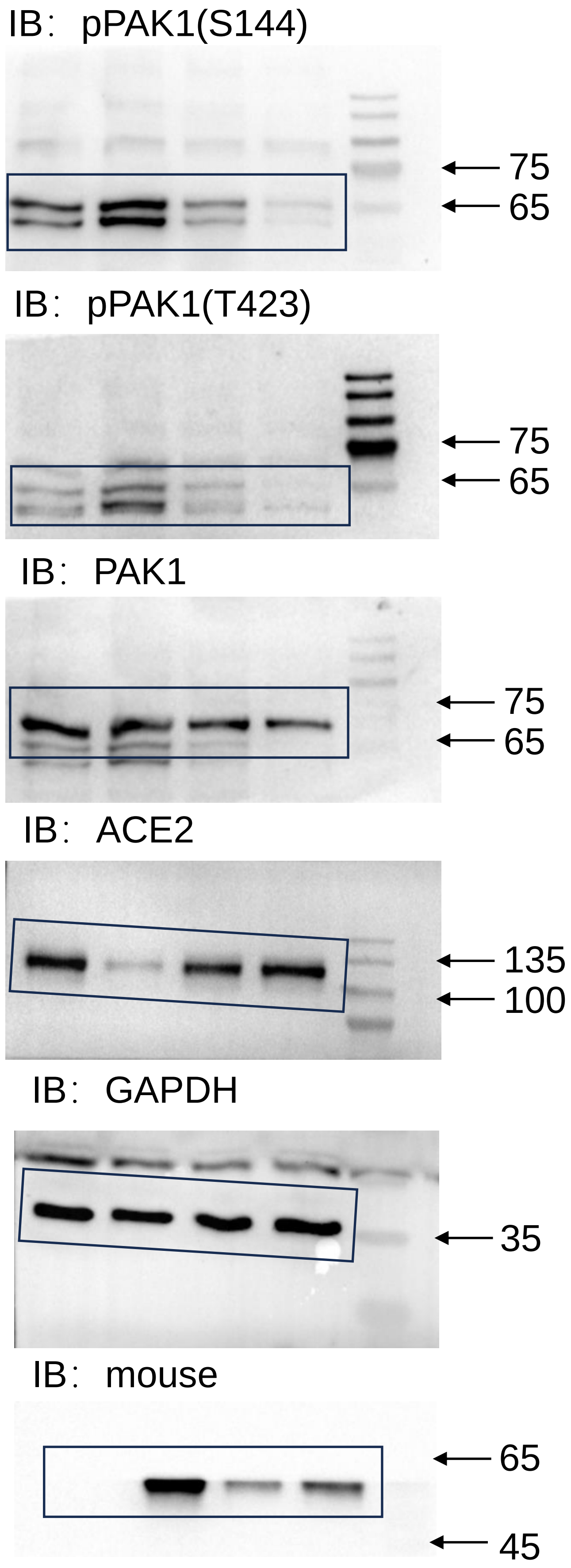
IB: pPAK1(S144)
75
65
IB: pPAK1(T423)
75
65
IB: PAK1
75
65
IB: ACE2
135
100
IB: GAPDH
35
IB: mouse
65
45
